# Design, synthesis and SAR exploration of tri-substituted 1,2,4-triazoles as inhibitors of the annexin A2–S100A10 protein interaction

**DOI:** 10.1016/j.bmc.2014.07.043

**Published:** 2014-10-01

**Authors:** Tummala R.K. Reddy, Chan Li, Xiaoxia Guo, Peter M. Fischer, Lodewijk V. Dekker

**Affiliations:** School of Pharmacy, Centre for Biomolecular Sciences, University of Nottingham, Nottingham NG7 2RD, United Kingdom

**Keywords:** S100 proteins, Annexins, Docking, Triazole

## Abstract

Recent target validation studies have shown that inhibition of the protein interaction between annexin A2 and the S100A10 protein may have potential therapeutic benefits in cancer. Virtual screening identified certain 3,4,5-trisubstituted 4*H*-1,2,4-triazoles as moderately potent inhibitors of this interaction. A series of analogues were synthesized based on the 1,2,4-triazole scaffold and were evaluated for inhibition of the annexin A2–S100A10 protein interaction in competitive binding assays. 2-[(5-{[(4,6-Dimethylpyrimidin-2-yl)sulfanyl]methyl}-4-(furan-2-ylmethyl)-4*H*-1,2,4-triazol-3-yl)sulfanyl]-*N*-[4-(propan-2-yl)phenyl]acetamide (**36**) showed improved potency and was shown to disrupt the native complex between annexin A2 and S100A10.

## Graphical abstract

**Figure:**
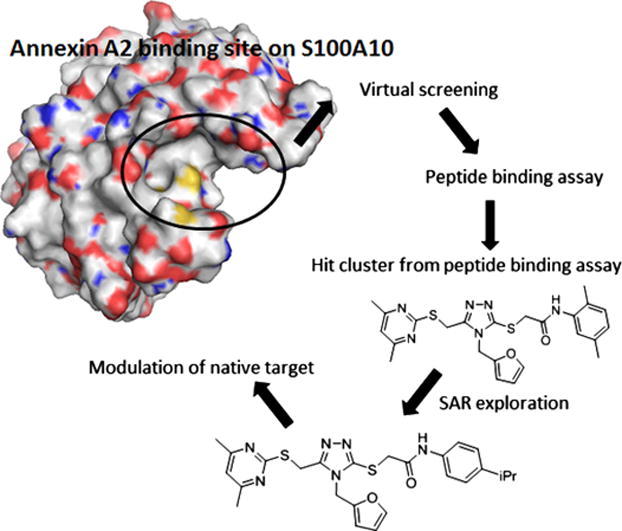

## 1 Introduction

Small molecule drug discovery has largely focused on drug targets within a fairly limited set of families of enzymes and receptors that offer tractable pharmacological space. Proteomics research has opened up a new, potentially very rich, target area consisting of the interacting faces of protein partners. Recent studies have provided evidence that protein–protein interactions could offer scope for small molecule intervention. For example, the interaction between Mdm2 and p53, and the interaction between Bcl2 and Bak have both been explored pharmacologically using small molecule inhibitors, which have subsequently shown promise as therapeutic agents.^1^, ^2^, ^3^ It is of interest that both p53 and Bak contain a short helical sequence that docks into a well-defined groove-like feature on the surface of the respective binding partners, which in both cases constitutes a small globular protein. This suggests that some protein interactions, characterised by these features, may be manipulated using small molecules.

S100 proteins constitute a family of small globular adaptor proteins that regulate cell functions by virtue of their capacity to interact with protein binding partners.^4^, ^5^ Annexins are important binding partners of S100 proteins^5^ and they contain a short helical sequence feature at the N-terminus that allows binding to several members of the S100 family. The S100A10 protein and annexin A2 (AnxA2) form a classic pairing in this way.^6^ The interaction between these proteins is very well characterised and shows similarities with the tractable protein interactions described above, in that a small helix docks into a deep well-defined binding crevice.^7^, ^8^ Both S100A10 and AnxA2 have been implicated in cell matrix invasion, cell movement and cell adhesion and as such play important roles in the regulation of therapeutically relevant processes such as vascular neo-angiogenesis and tumour cell metastastis.^9^, ^10^, ^11^, ^12^

Using receptor-guided random docking approaches and biochemical screening, we have previously identified several clusters of small molecule inhibitors of this protein interaction.^13^ Here we describe the optimization of a cluster of substituted 1,2,4 triazole compounds emanating from this docking strategy to achieve inhibitors with improved potency.

## 2 Results and discussion

### 2.1 Structure-based screening

**Figure 1:**
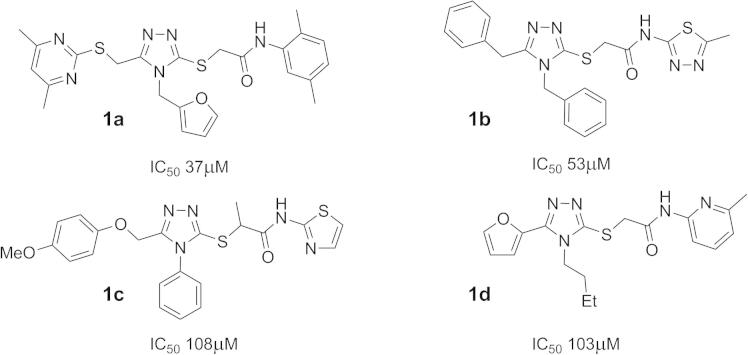
Chemical structures and IC_50_ values of the active compounds **1a**–**d** identified through virtual screening.^13^

**Figure 2:**
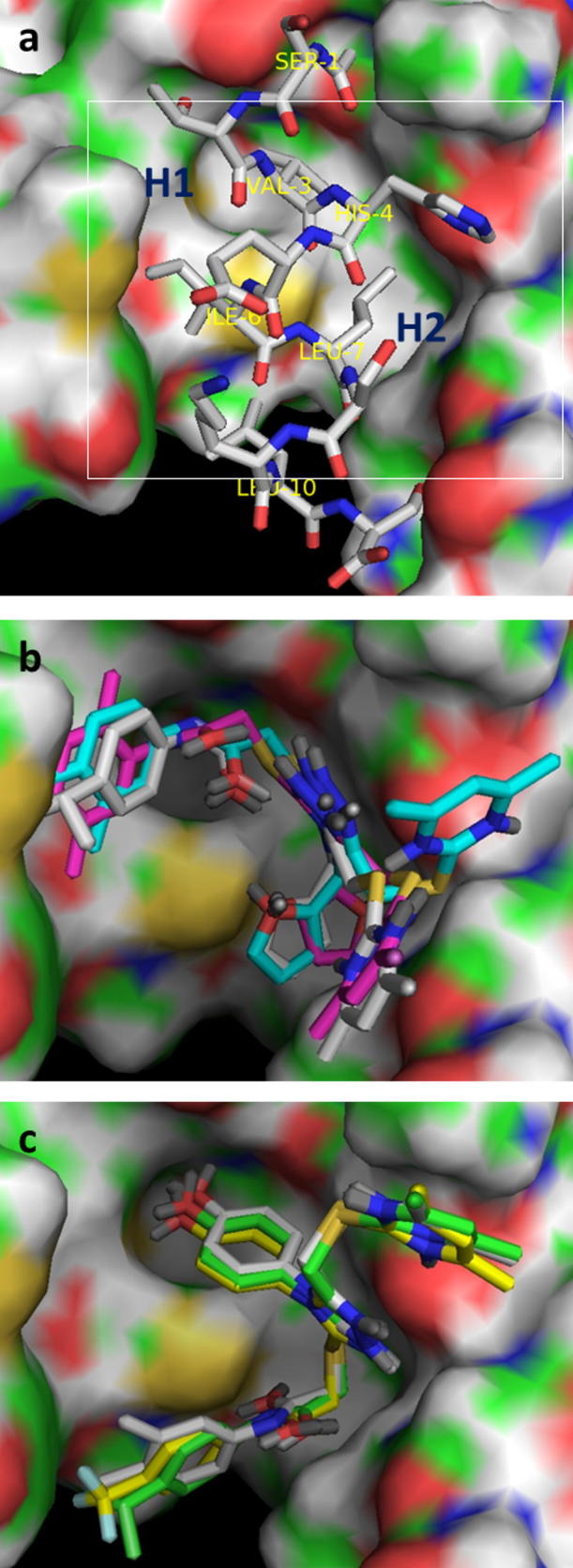
(a) Binding mode of the AnxA2 N-terminal peptide (represented as sticks; C: grey, N: blue, O: red) within the binding pocket on S100A10 (molecular surface C: green, N: blue, O: red, S: yellow, H: white). Hydrophobic regions of the binding pocket (H1 and H2) are shown. The white rectangle represents the area of the binding pocket used to visualise the docked poses in panels b and c. (b) Predicted binding modes of compounds **1a** (C: magenta, N: dark blue, O: red, S: yellow, lone pairs: dark grey), **35** (C: light blue), and **36** (C: light grey). Hydrogens have been removed. (c) Predicted binding modes of compounds **48** (C: green), **52** (C: bright yellow, F: light blue), and **43** (C: light grey). Methyl moiety on OMe groups projects downwards and is obscured in the figure. Figure created in Pymol (DeLano).

**Figure 3:**
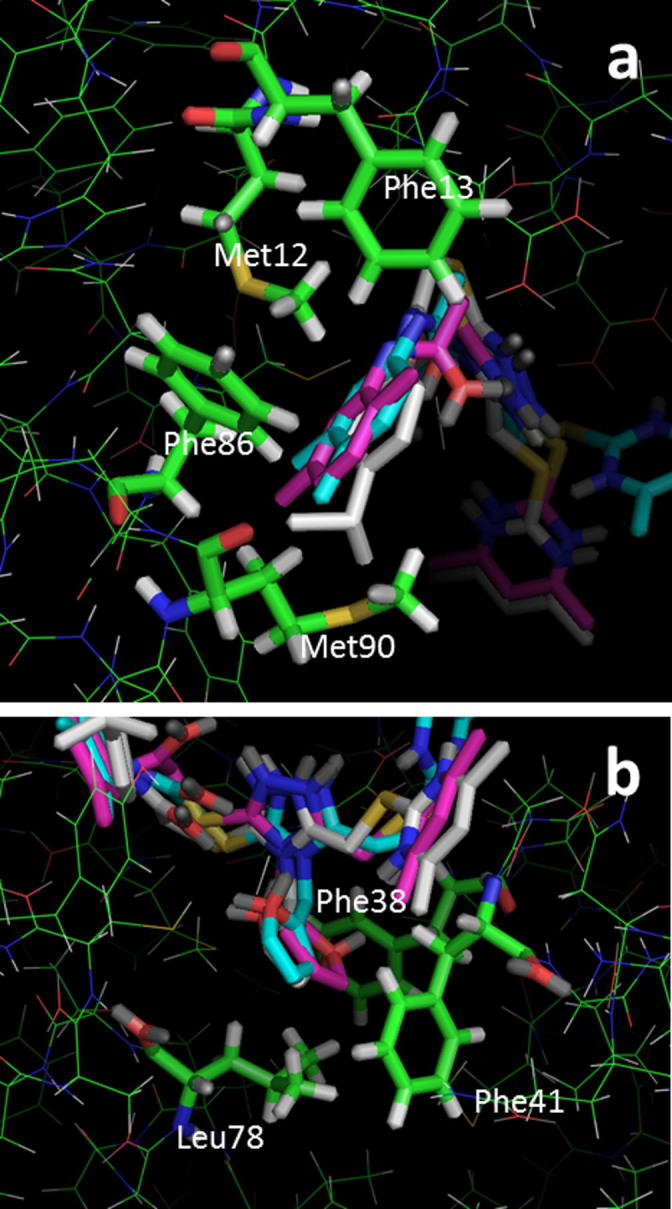
Hydrophobic residues in the S100A10 dimer (represented as sticks C: green, N: blue, O: red, S: yellow, H: white) creating binding areas H1 (a) and H2 (b) for the substituted phenyl ring (a) and pendant furfuryl ring (b) of compounds **1a**, **35** and **36**. Compounds are coloured as in Figure 2b. Figure created in Pymol (DeLano).

We have previously reported the in silico screening of 0.7 million compounds using the AnxA2 binding pocket of S100A10 as a receptor.^13^ Following detailed analysis of the binding poses, and biochemical assessment of the ability of the top scoring compounds to inhibit the interaction between S100A10 and the AnxA2 N-terminus, 29 potential inhibitory compounds were identified, which were distributed over 10 different clusters. A cluster of four trisubstituted 1,2,4-triazoles showed a degree of inhibitory activity that appeared to relate to their chemical structure (**1a**–**d**; Fig. 1). This was of interest since we have previously used ligand-guided screening methods to identify similar 1,2,4-triazoles as moderately potent inhibitors of this protein interaction.^14^ Analysis of the binding mode of **1a**, predicted using the GOLD programme^15^ (Fig. 2b), suggested that it lies across the binding groove occupied by the cognate AnxA2 N-terminus (Fig. 2a), thereby inhibiting access of the latter. Compound **1a** interacts mainly with hydrophobic areas of this binding groove (identified as H1 and H2 in Fig. 2b). The orientation of the substituted phenyl moiety in compound **1a** is governed by hydrophobic CH–π stacking interactions mediated by Met12 in the hydrophobic region H1. Both Phe86 and Phe13 are also predicted to contribute to the binding of this group, through hydrophobic and van der Waals interactions (Fig. 3a). The lower potency of the compounds with more polar heterocyclic systems at the position of this phenyl moiety (Fig. 1, **1b**–**d**) is compatible with the hypothesis that a hydrophobic binding region accommodates this ring system. The 1,2,4-triazole core of **1a** is positioned at the centre of the AnxA2 binding groove, whereas the furfuryl substituent at the triazole N4-position is located towards the H2 region and is predicted to make hydrophobic interactions with Leu78, Phe38 and Phe41 (Fig. 3b). From this binding mode analysis it appears that the two hydrophobic regions H1 and H2 play a dominant role in the inhibitory potency of compound **1a**. The potency of **1a** (IC_50_ = 37 μM) was low compared that of the cognate AnxA2(1–14) peptide ligand (IC_50_ = 1.2 μM), but similar to that previously observed for related 1,2,4-triazoles, which lacked the acetamide-linked aromatic ring system.^14^ Here we have investigated structure–activity relationships of this series of compounds, focusing in particular on the contribution of this aromatic system to inhibition of the protein interaction with the aim of gaining improvements in the potency of these compounds.

### 2.2 Chemical synthesis

**Scheme 1:**
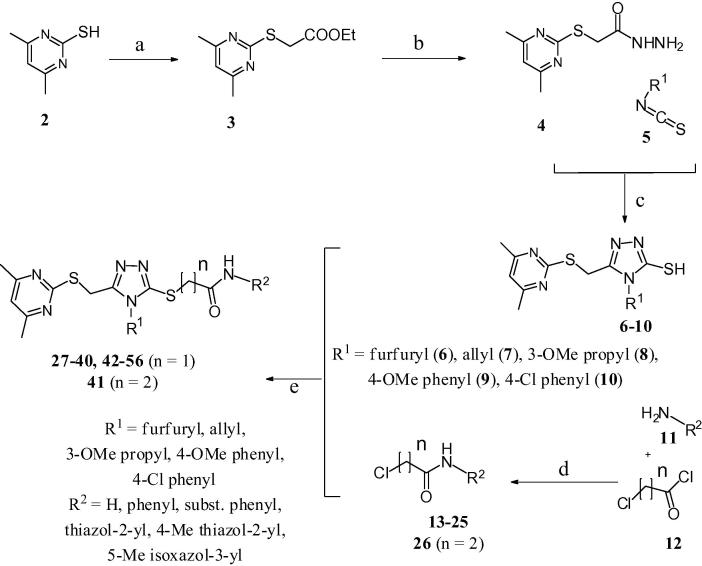
Synthetic route for 3,4,5-tri-substituted 1,2,4-triazoles. Reagents and conditions: (a) bromoethyl acetate, NaOAc in EtOH, reflux for 1 h; (b) NH_2_–NH_2_·H_2_O, reflux overnight; (c) (i) EtOH, reflux overnight; (ii) 1 M aq NaOH, 45 °C, 45 min then 10% aq HCl; (d) CH_2_Cl_2_, Et_3_N, RT, overnight; (e) K_2_CO_3_, DMF, 45 °C, overnight, then H_2_O.

*Compounds **1a**, **27**–**56**.* The substituted ethyl acetate **3** (Scheme 1) was prepared by reacting 4,6-dimethyl-pyrimidine-2-thiol **2** with bromoethyl acetate in the presence of sodium acetate as a base in ethanol. Subsequent reaction of ester **3** with excess hydrazine monohydrate yielded acyl hydrazide **4**. Condensation of the latter with various substituted aromatic or aliphatic isothiocyanates **5** in ethanol, followed by base-catalysed cyclization, resulted in the formation of substituted 1,2,4-triazoles (**6**–**10**). Chloro-*N*-aryl-acetamides **13**–**26** were prepared by reacting substituted aryl amines **11** with 2-chloroactyl or propionyl chlorides **12** in the presence of Et_3_N as a base.

Analogues of **1a** were synthesized using a 12-well Radley’s parallel synthesizer. 3-Mercapto-1,2,4-triazole derivatives **6**–**10** were treated with chloro-*N*-aryl-acetamide derivatives **13**–**26** in the presence of inorganic base in DMF to afford the desired compounds **27**–**56** (Scheme 1) in moderate to good yields.^16^, ^17^, ^18^, ^19^, ^20^, ^21^, ^22^

**Scheme 2:**
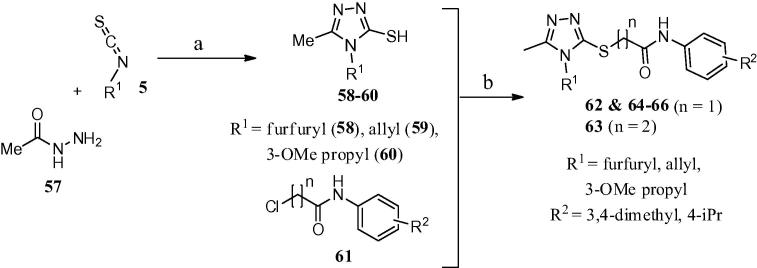
Synthetic route for 5-methyl-1,2,4-triazole analogues. Reagents and conditions: (a) (i) EtOH, reflux, overnight; (ii) 1 M aq NaOH, 45 °C, 90 min then 10% aq HCl; (b) K_2_CO_3_, DMF, 40 °C, overnight, then H_2_O.

*Compounds **62**–**66**.* Condensation of the acetohydrazide **57** (Scheme 2) with aromatic or aliphatic isothiocyanates **5** in ethanol followed by base-catalysed cyclization afforded the required substituted 3-mercapto-5-methyl-1,2,4-triazoles **58**–**60**. Finally, the target 5-methyl-1,2,4-triazoles **62**–**66** were prepared by reacting substituted 3-mercapto-5-methyl-1,2,4-triazoles **58**–**60** with chloro-*N*-aryl-acetamides **61** in the presence of potassium carbonate as a base in DMF.^23^, ^24^, ^25^, ^26^, ^27^, ^28^, ^29^, ^30^, ^31^, ^32^, ^33^

### 2.3 Structure–activity relationships (SARs)

#### 2.3.1 Nature of the acetamide-linked ring system

**Figure 4:**
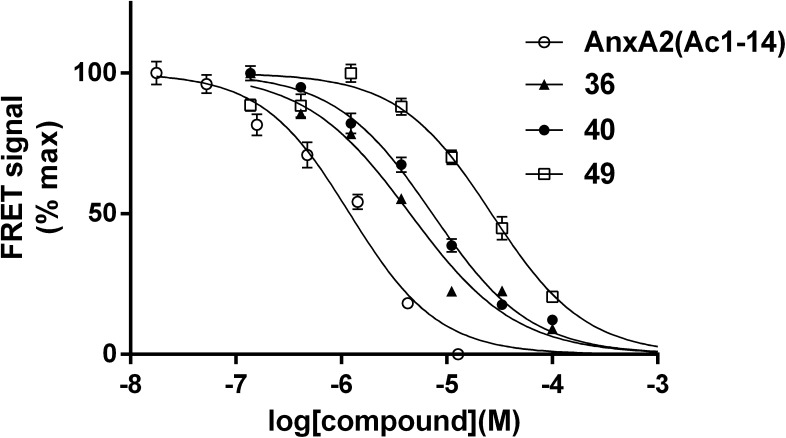
Inhibition of the interaction between the AnxA2 N-terminus and S100A10 by Non-labelled AnxA2(Ac1–14) peptide (○) or compounds **36** (▴), **40** (●), and **49** (□).

Taking compound **1a** as starting point, we replaced the substituted phenyl ring side chain with an aliphatic amide side chain to yield **27**. The reduction in potency associated with this modification suggested the importance of the phenyl ring for binding (see Fig. 4 for typical IC_50_ curves). Replacement of the substituted phenyl ring with the aromatic but more polar thiazole (**28**) resulted in lower inhibitory activity comparable to what was observed above for compound **1b**. Introduction of a methyl substituent onto the thiazole reversed the potency drop (**29**). A similar pattern was observed for the related, methyl-substituted isoxazole ring at this position (**30**). The lower potency of **28** can be explained by postulating that binding mainly relies on hydrophobic interactions, in keeping with the predicted model in which the phenyl group of **1a** occupies the hydrophobic H1 region of the AnxA2 binding groove (Figs. 2b and 3a). We therefore investigated if introduction of alternative hydrophobic substituents into the ring system would result in better interactions (Table 1).

**Table 1:** SARs of 2-[5-(4,6-dimethyl-pyrimidin-2-ylsulfanylmethyl)-4-furan-2-ylmethyl-4*H*-[1,2,4]triazol-3-ylsulfanyl]-*N*-substituted (*R*)-acetamide analogues (**1a**–**d** and **27**–**40**). Data are based upon 12 observations

**Figure:**
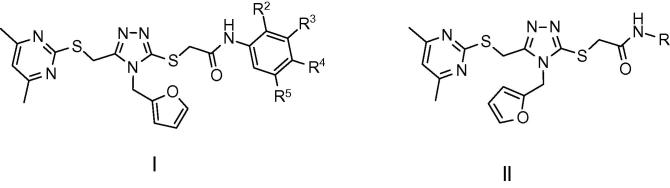

Compd	Type	R^2^	R^3^	R^4^	R^5^	R	pIC_50_ ± SE	IC_50_ (μM)
Peptide							5.92 ± 0.04	1.2
⁎**1a**	I	Me	H	H	Me		4.42 ± 0.08	37
⁎**1b**							4.28 ± 0.06	53
⁎**1c**							3.96 ± 0.14	108
⁎**1d**							3.98 ± 0.10	103
**27**	II	—	—	—	—	H	3.75 ± 0.15	178
**28**	II	—	—	—	—	Thiazol-2-yl	3.89 ± 0.06	129
**29**	II	—	—	—	—	4-Me-thiazol-2-yl	4.53 ± 0.06	30
**30**	II	—	—	—	—	5-Me-isoxazol-3-yl	4.37 ± 0.07	43
**31**	I	H	H	H	H	—	4.63 ± 0.04	23
**32**	I	H	Me	H	H	—	4.89 ± 0.04	13
**33**	I	H	H	Me	H	—	4.96 ± 0.04	11
**34**	I	Me	H	Me	H	—	4.83 ± 0.04	15
**35**	I	H	Me	Me	H	—	5.02 ± 0.04	10
**36**	I	H	H	iPr	H	—	5.35 ± 0.04	4
**37**	I	H	H	CF_3_	H	—	4.55 ± 0.07	28
**38**	I	H	CF_3_	H	CF_3_	—	5.05 ± 0.03	9
**39**	I	H	H	Cl	H	—	4.76 ± 0.03	17
**40**	I	H	H	Br	H	—	5.14 ± 0.03	7

⁎ Compounds **1a**–**d** were purchased from Asinex.

#### 2.3.2 Acetamide-linked phenyl ring substitutions

An unsubstituted phenyl ring (**31**) was not associated with a reduction in potency, however, repositioning the two methyl substituents on the phenyl group increased potency, suggesting that the substitution pattern is in fact important. Compared to an unsubstituted ring (**31**), introduction of a single methyl group at either the 3- or 4-position was associated with a small increase in potency (**32** and **33**). The 2,4-dimethyl substituted analogue **34** displayed similar potency, while the 3,4-dimethyl substitution in **35** resulted in a moderate increase in potency. Analysis of the predicted binding mode of compound **35** (Fig. 2b) suggested that the orientation of the 3,4-dimethylphenyl moiety is governed by hydrophobic interactions and causes a change in the location of the 4,6-dimethylpyrimidine moiety, which differs from that observed for compound **1a** (Fig. 2b) in that it is flipped away into a more hydrophilic region. As a result it is involved in more polar interactions with Gly40 and Ser2. Introduction of a bulkier hydrophobic isopropyl substitution at the *para*-position of the phenyl ring (**36**) yielded a compound with an IC_50_ value of 4 μM, which is almost 10-fold greater than the initial hit compound **1a** and comparable to that of the cognate peptidic ligand (IC_50_ = 1 μM). This is highly reminiscent of the SAR observed for a distinct series of inhibitors of this target we identified previously.^13^ A predicted docking solution of **36** (Fig. 2b) suggested that this compound had a similar binding pose as **1a**, with the *para*-isopropyl group projecting towards the H1 pocket likely to be the main reason for its improved activity. We also tested the effect of hydrophobic electron-withdrawing substitutions on the phenyl ring. Although a CF_3_ substitution at the para position (**37**) did not provide any advantage over the unsubstituted ring system in terms of potency, the 3,5-di-CF_3_ analogue **38** was comparatively potent. Introduction of a *para-*chloro phenyl substituent (**39**) did not result in higher potency, however, the *para*-bromo derivative **40** was 5-fold more potent than compound **1a**.

#### 2.3.3 Triazole N4 substituents

We next turned our attention to N4 substituents (R^1^) of the 1,2,4-triazole scaffold (Table 2). For compound **35**, the furfuryl substituent at this position is predicted to project towards the hydrophobic region H2 (Fig. 3b). The specific relevance of the hydrophobic, aromatic and electronic characteristics of this group were investigated. Substitution of the furfuryl moiety of compound **35** with a hydrophobic allyl substituent retaining π electronic characteristics (**41**), a mixed aliphatic and polar (3-methoxypropyl) substituent (**42**), or an aromatic polar (4-methoxyphenyl) substituent (**43**) and aromatic chloro (4-chlorophenyl) substituent (**44**), all resulted in a reduction of potency, suggesting that the furfuryl group possessed a specific combination of geometric and chemical properties relevant to support binding. Using compound **35** as the starting point, extension of the sulfanyl side chain by addition of a methylene group (**45**) did not appear to be advantageous in terms of activity and was not explored further.

**Table 2:** SARs of 3,4,5-trisubstituted-1,2,4-triazole analogues (**35**–**37** and **41**–**56**). Data are based upon 12 observations

**Figure:**
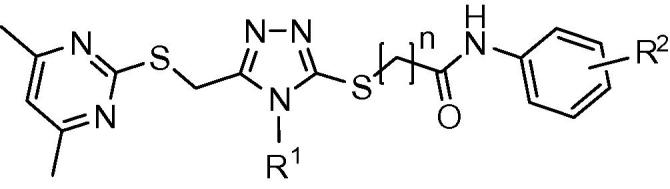

Compd	*n*	R^2^	R^1^	pIC_50_ ± SE	IC_50_ (μM)
**35**	1	3,4-Dimethyl	Furfuryl	5.02 ± 0.04	10
**41**	1	3,4-Dimethyl	Allyl	4.56 ± 0.03	27
**42**	1	3,4-Dimethyl	3-OMe-propyl	4.49 ± 0.04	32
**43**	1	3,4-Dimethyl	4-OMe-phenyl	4.29 ± 0.09	52
**44**	1	3,4-Dimethyl	4-Cl-phenyl	4.23 ± 0.08	59
**45**	2	3,4-Dimethyl	Furfuryl	4.62 ± 0.03	24
**36**	1	4-*i*Pr	Furfuryl	5.35 ± 0.04	4
**46**	1	4-*i*Pr	Allyl	5.01 ± 0.03	10
**47**	1	4-*i*Pr	3-OMe-propyl	5.13 ± 0.04	7
**48**	1	4-*i*Pr	4-OMe-phenyl	4.82 ± 0.02	15
**49**	1	4-*i*Pr	4-Cl-phenyl	4.58 ± 0.04	27
**37**	1	4-CF_3_	Furfuryl	4.55 ± 0.07	28
**50**	1	4-CF_3_	Allyl	5.08 ± 0.04	8
**51**	1	4-CF_3_	3-OMe-propyl	5.02 ± 0.04	10
**52**	1	4-CF_3_	4-OMe-phenyl	5.35 ± 0.04	4
**53**	1	4-CF_3_	4-Cl-phenyl	5.07 ± 0.03	8
**54**	1	3,5-Di-CF_3_	4-OMe-phenyl	5.14 ± 0.04	7
**55**	1	4-Cl	4-OMe-phenyl	4.65 ± 0.05	22
**56**	1	4-MeO	4-OMe-phenyl	3.81 ± 0.03	154

Following the substitution patterns of the most potent compound (**36**) in our series, we combined the 4-(isopropyl)phenyl substitution with allyl (**46**), 3-methoxypropyl (**47**), 4-methoxyphenyl (**48**) and 4-chlorophenyl (**49**) at the triazole N4 position. The resulting isopropyl derivatives were all somewhat less active than **36** itself but were each individually more potent than the corresponding compound from the 3,4-dimethyl series that was based on compound **35**. Thus in the context of hydrophobic electron-donating substitutions at the phenyl ring, of the groups tested, the N4 furfuryl group appeared to be optimal.

A rather different picture emerged upon similar replacements for compound **37**, which contains a CF_3_ moiety at the *para*-position of the phenyl ring. Allyl (**50**), 3-methocypropyl (**51**), 4-methoxyphenyl (**52**) and 4-chlorophenyl (**53**) substitutions resulted in increased potency compared to **37**. The methoxyphenyl derivative **52** was the most potent compound in this group and is 10-fold more potent than the starting compound **1a**. It was also more potent than the triazole-N4-substituted 4-methoxyphenyl derivatives containing electron-donating isopropyl (**48**) or 3,4-dimethyl (**43**) groups on the acetamide-linked phenyl ring. Alternative replacements at this phenyl ring did not improve the potency achieved with that of compound **52**. The 3,5-diCF_3_ phenyl derivative **54** was equipotent with **52** while the 4-chlorophenyl derivative **55** and the 4-methoxy derivative **56** were less potent. Weak resonance donating properties of the halogen may be relevant in this context, as could be the electron-donating properties of the methoxy group. Overall, the 4-methoxyphenyl group at N4 appeared optimal for compounds that contained a hydrophobic electron-withdrawing group at the phenyl ring, while substitutions that increase ring electron density were not favoured in this context.

Interestingly, docking studies of the N4 4-methoxyphenyl derivatives (**43**, **48**, **52** and **54**–**56**) showed that most of the compounds adopted an alternative binding pose compared to that of the N4 furfuryl compounds (Fig. 2c). According to this binding pose, compounds stretch across most of the binding area occupied by the N-terminus of the cognate AnxA2 ligand (Fig. 2c). This binding pose could be due to the introduction of bulk (*para*-substituted methoxyphenyl groups) at the N4-position of the triazole ring. The 4-methoxyphenyl substituent of the compounds appears to mimic the interactions of the Val3 side chain of the AnxA2 peptide in the crystal structure (Fig. 2a and c). The presence of AnxA2-Val3 is essential for binding to S100A10 since its substitution leads to substantial loss of binding.^34^ In all these compounds, the 4,6-dimethylpyrimidine moiety extends into the hydrophilic region, similar to what was predicted for compound **35** (Fig. 2b), whereas the *para*-substituted phenyl moiety is involved in hydrophobic interactions with the far end of the H1 binding pocket. In the case of compound **52** the *para*-CF_3_-phenyl moiety makes good van der Waals and hydrophobic interactions (Fig. 2c), whereas the *para*-(isopropyl)phenyl moiety of compound **48** (Fig. 2c) is involved in weaker hydrophobic interactions than the corresponding *para*-CF_3_ derivative **52**. The low potency of compound **43** could not be justified based on an alternative binding mode as the 3,4-dimethylphenyl moiety appears to form good hydrophobic and van der Waals interactions with the far end of the H1 binding pocket.

#### 2.3.4 Sulfanyl side chain

Finally, we examined the importance of the 4,6-dimethylpyrimidin-2-ylsulfanyl side chain on inhibitory potency (Table 3). Inspection of the predicted binding pose of compound **1a** shows that the side chain in question interacts with the comparatively hydrophilic region of the binding pocket (Figs. 2b and 3a). Truncation of the 4,6-dimethylpyrimidin-2-ylsulfanyl side chain of compound **35** to the corresponding methyl derivative **62** resulted in complete loss of activity. Extending the side chain of compound **62** by an additional methylene group (**63**) also abolished activity. Truncation of the 4,6-dimethylpyrimidin-2-ylsulfanyl side chain of compound **36** to give the corresponding methyl derivative **64** resulted in a 10-fold loss of activity, whereas in the case of compounds **46** and **47** similar truncation to methyl derivatives **65** and **66** resulted in 14- and 23-fold lower activity, respectively. Thus the 4,6-dimethylpyrimidin-2-ylsulfanyl side chain makes an important contribution to the inhibitory activity of these compounds.

**Table 3:** SARs of 5-methyl-4-substituted (*R*^2^)-4*H*-[1,2,4]triazole analogues (**62**–**66**). Data are based upon 12 observations

**Figure:**
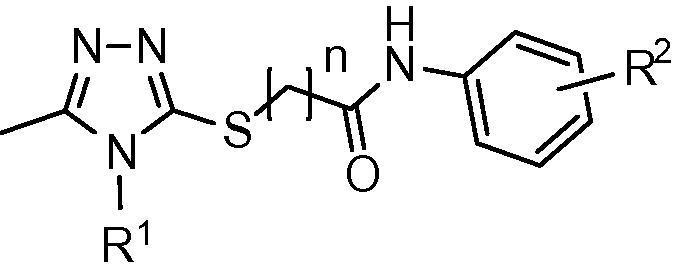

Compd	*n*	R^2^	R^1^	pIC_50_ ± SE	IC_50_ (μM)
**62**	1	3,4-Dimethyl	Furfuryl	2.32 ± 0.48	>1000
**63**	2	3,4-Dimethyl	Furfuryl	2.95 ± 0.26	>1000
**64**	1	4-*i*Pr	Furfuryl	4.40 ± 0.03	40
**65**	1	4-*i*Pr	Allyl	3.86 ± 0.11	138
**66**	1	4-*i*Pr	3-OMe-propyl	3.79 ± 0.14	164

### 2.4 Inhibition of native complex

**Figure 5:**
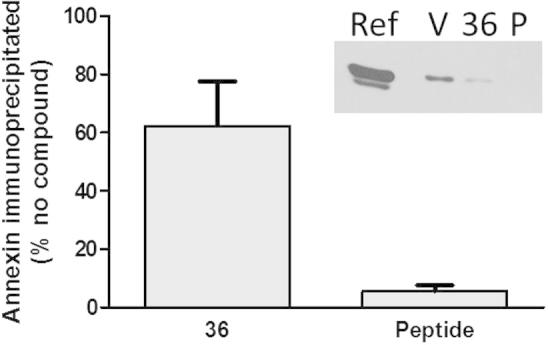
Co-immunoprecipitation of AnxA2 with S100A10. S100A10 was immunoprecipitated from cell lysates and co-immunoprecipitation of AnxA2 was assessed by Western blotting. The amount of AnxA2 in the S100A10 immunoprecipitate was quantified using Scion Image Software and expressed as a percentage of the vehicle-treated sample (*n* = 4 ± SE). Inset shows a typical Western blot labelled as follows: ref, total lysate reference indicating the AnxA2 position on the gel; V, 36, P, immunoprecipitates of lysates preincubated with vehicle, compound **36** or AnxA2(1–14) peptide, respectively.

Although it is clear that the tri-substituted 1,2,4 triazoles act as inhibitors of the binding of the N-terminus of AnxA2 to S100A10, it is necessary to confirm that the native complex of the two full-length proteins, AnxA2 and S100A10, is also inhibited. Interaction between the native proteins can be measured by co-immunoprecipitation as shown in Figure 5 and previously.^13^ AnxA2 is detected by Western blotting after immunoprecipitation of the S100A10 protein, suggesting that binding occurs between the two proteins. In the presence of the isolated synthetic peptide AnxA2(1–14), the amount of AnxA2 in the S100A10 immunoprecipitate is reduced, due to it competing with the binding between native AnxA2 and S100A10. In the presence of compound **36**, the amount of AnxA2 in the immunoprecipitate is also reduced, indicating that it does indeed inhibit the native complex.

## 3 Conclusions

Analysis of the molecular interactions of compound **1a** shows that the hydrophobic pockets H1 and H2 of the S100A10 receptor play a crucial role in the affinity of these compounds. Complex interactions appear to occur between the N4- and the acetamide-linked ring systems, such that dependent on the nature of the N4 substituent, the hydrophobic character and the electron density on the acetamide-linked ring is correlated with inhibition patterns. Additionally, the 4,6-dimethylpyrimidin-2-ylsulfanyl side chain appears to be important for the activity, and this needs further exploration.

Compounds **36** and **52** are the most potent in present compound series with 10-fold increase in activity compared to the initial hit compound **1a**. They also displayed higher activity than a set of triazole analogues that lack an acetamide-linked ring that we have reported on previously.^14^ Compounds **36** and **52**, and various analogues described here, could thus be used as probes further to understand the molecular interactions between S100A10 and AnxA2 and may also serve as potential starting point for further optimization.

## 4 Experimental

### 4.1 Protein structure preparation

SYBYL-X 1.0 biopolymer was used for the preparation of the S100A10–AnxA2 N-terminal peptide complex (PDB ID: 1BT6). The chain termini (Lys91, Pro1 and Ser11 of AnxA2) were charged. Hydrogen atoms were added. AMBER 7 FF99 charges were assigned to the complex. The side chain amides of Gln45 and Gln60 were re-oriented to maximize hydrogen bonding. Powell’s energy minimization of the complex was achieved by a stepwise manner using AMBER 7 FF99 parameters. Initial optimization was not performed, energy gradient of 0.5 kcal/mol was maintained and a maximum of 5000 iterations were used. A centroid was defined by a set of atoms within 5 Å of the AnxA2 ligand peptide and the peptide was extracted from the complex. Water molecules were deleted before docking.

### 4.2 GOLD (v3.0.1) docking

Docking of each ligand was performed in 10 different genetic algorithm (GA) runs, and for each of these a maximum number of 100,000 GA operations were performed on a single population of 100 individuals. Early termination of docking runs was allowed if the top three solutions were within 1.5 Å RMSD of each other. Crossover, mutation, migration (95, 95 and 10, respectively), hydrogen bonding (4.0 Å), and van der Waals (2.5 Å) parameters were set as default value throughout the docking. A binding site radius of 12 Å was found to be optimal. Flipping was not allowed for those ligands that have ring-NHR and ring-NHR^1^R^2^ groups in order to avoid the addition of large torsional energy penalties to the total fitness scores.

### 4.3 Fluorescence screening assay

Assessment of compound activity was performed as described.^35^ Briefly, a Cy5-labelled S100A10 tracer was developed and binding of a Cy3-labelled AnxA2(Ac1–14) peptide ligand was assessed using a fluorescence resonance energy transfer readout. Assays were carried out in Nunc black non-treated 384-well plates at 20 °C in 50 μL buffer C. All incubations were performed in quadruplicate. Compounds, peptide and buffer controls were added to the wells in a 10 μL volume in 5% DMSO. Cy5-labelled S100A10 tracer (407 nM) and Cy3-labelled AnxA2(Ac1–14) peptide ligand (1.33 μM) were pre-incubated for 5 min at 20 °C and 40 μL of the preformed complex was then added to the wells and mixed for 10 s to yield a final DMSO concentration of 1%. After 5 min incubation at 20 °C, readings were taken on a Perkin Elmer Envision plate reader by excitation at 488 nm and emission at 695 nm. Fluorescence resonance energy transfer (FRET) was calculated by subtracting the sum of the fluorescence emission of S100A10-Cy5 and AA2(1–14)-Cy3 individually from that measured for the co-incubated partners. Compound binding was calculated as percentage of non-treated control and data were analysed by non-linear regression (dose response—variable slope with bottom and top parameters fixed at 0% and 100%, respectively) using Graphpad Prism Software to determine the IC_50_ of binding (primary assay).

### 4.4 Immunoprecipitation assay

Human MDA-MB-231 breast cancer cells were extracted in 50 mM Tris–HCl (pH 8.0) buffer containing 150 mM sodium chloride and 1% (v/v) NP-40. Lysates were centrifuged at 17,000×*g* and incubated at 4 °C for 16 h with 10 μL S100A10 antibody (BD transduction Laboratories) and 50 μL protein A/G agarose (Alpha Diagnostic International Inc). Protein A/G agarose was then recovered by centrifugation at 2400×*g*, washed three times with 10 mM phosphate buffer (pH 7.4) containing 2.7 mM potassium chloride and 137 mM sodium chloride, resuspended in 50 μL of 160 mM Tris–HCl (pH 6.8) buffer containing 4% (w/v) sodium lauryl sulfate, 20% (v/v) glycerol, 0.04% (w/v) bromophenol blue and 10% (v/v) 2-mercaptoethanol, boiled at 99 °C for 10 min and centrifuged at 17,000×*g* for 10 min. The supernatant was then analyzed by SDS–PAGE after which the gel was transferred to a nitrocellulose filter. The filter was incubated with an AnxA2 monoclonal antibody (1:3000; BD Transduction Laboratories) followed by incubation with an anti-mouse horseradish peroxidase IgG conjugate (1:5000; GE Healthcare) and then developed using the ECL detection reagent (GE Healthcare).

### 4.5 Synthesis

All reagents were purchased directly from commercial sources and were used as supplied, unless otherwise stated. Accurate mass and nominal mass measurements were performed using a Waters 2795-Micromass LCT electrospray mass spectrometer. All NMR spectra were recorded in deutero-DMSO in 5 mm tubes, with trimethylsilane as an internal standard, using a Bruker ACS-120 instrument at 400 MHz (^1^H NMR). Thin layer chromatography was performed using aluminium-backed silica gel 60 plates (0.20 mm layer), the ascending technique was used with a variety of solvents. Visualization was by UV light at either 254 or 365 nm.

#### 4.5.1 (4,6-Dimethyl-pyrimidin-2-ylsulfanyl)-acetic acid ethyl ester (**3**)

To a solution of **2** (14.2 g, 100 mmol) in EtOH (190 mL) was added NaOAc (12.3 g, 150 mmol) and ethyl bromoacetate (11.3 mL, 100 mmol). The mixture was heated under reflux for 60 min and EtOH was then evaporated. The residue was diluted with H_2_O and extracted with EtOAc. The extract was dried over Na_2_SO_4_, filtered, and concentrated under vacuum to afford **3** as a yellow oil (15.5 g, 69%). *m*/*z* (ES), found 227.0821 (C_10_H_15_N_2_O_2_S [M+H]^+^) requires 227.2954; *δ*_H_/ppm (400 MHz, *d*^6^-DMSO): 6.97 (1H, s, Ar-H), 4.10 (2H, q, *J* = 7.1, CH_2_), 3.94 (2H, s, CH_2_), 2.33 (6H, s, 2CH_3_), 1.18 (3H, t, *J* = 7.1, CH_3_).

#### 4.5.2 (4,6-Dimethyl-pyrimidin-2-ylsulfanyl)-acetic acid hydrazide (**4**)

To a solution of **3** (12.3 g, 54.3 mmol) in EtOH (170 mL) was added NH_2_NH_2_·H_2_O (16.1 mL, 32 mmol) and the mixture was heated under reflux overnight. The reaction was cooled and concentrated under vacuum. The pale white precipitate obtained was washed with petroleum ether (PE)–EtOH (3:1) and then dried under vacuum to afford **4** as a pale yellow powder (9.7 g, 84%). *m*/*z* (ES), found 213.0846 (C_8_H_13_N_4_OS [M+H]^+^) requires 213.0732; *δ*_H_/ppm (400 MHz, *d*^6^-DMSO): 9.21 (1H, s, NH), 6.96 (1H, s, Ar-H), 4.17 (2H, br s, NH_2_), 3.79 (2H, s, CH_2_), 2.34 (6H, s, 2CH_3_).

#### 4.5.3 5-(4,6-Dimethyl-pyrimidin-2-ylsulfanylmethyl)-4-furan-2-ylmethyl-4*H*-[1,2,4]triazole-3-thiol (**6**)

To a solution of **4** (5.0 g, 23.6 mmol) in EtOH (175 mL) was added furfuryl isothiocyanate (3.4 mL, 23.6 mmol) and the mixture was heated under reflux overnight. The reaction was cooled and concentrated under vacuum. The white precipitate obtained was washed with PE–EtOH (1:1). To the suspension was added 2 M aq NaOH (40 mL) and the mixture was heated under reflux for 3 h before cooling. The reaction was neutralized with 10% aq HCl and was extracted with EtOAc. The organic layer was dried over Na_2_SO_4_, filtered, and concentrated. The resulting yellow solid was recrystallized from EtOH to afford **6** as an off-white powder (2.4 g, 30%). *m*/*z* (ES), found 332.0606 (C_14_H_14_N_5_OS_2_ [M−H]^−^) requires 332.0718; *δ*_H_/ppm (400 MHz, *d*^6^-DMSO): 13.72 (1H, s, NH of thione tautomer), 7.63 (1H, t, *J* = 1.3/1.4, Ar-H), 6.98 (1H, s, Ar-H), 6.46–6.41 (2H, m, Ar-H), 5.37 (2H, s, CH_2_), 4.53 (2H, s, CH_2_), 2.31 (6H, s, 2CH_3_).

#### 4.5.4 4-Allyl-5-(4,6-dimethyl-pyrimidin-2-ylsulfanylmethyl)-4*H*-[1,2,4]triazole-3-thiol (**7**)

To a solution of **4** (4.5 g, 21.2 mmol) in EtOH (150 mL) was added allyl isothiocyanate (2.2 mL, 21.2 mmol) and the reaction mixture was heated under reflux overnight. It was cooled and the white precipitate formed was collected by filtration and was washed with EtOH. To the suspension was added 1 M aq NaOH (25 mL) and the mixture was heated at 45 °C for 90 min before cooling. The reaction was neutralized with 10% aq HCl. The yellow precipitate formed was collected by filtration, washed with H_2_O, followed by EtOH, and then vacuum dried to afford **7** as a pale white powder (2.9 g, 47%). *m*/*z* (ES), found 292.0616 (C_12_H_14_N_5_S_2_ [M−H]^−^) requires 292.0769; *δ*_H_/ppm (400 MHz, *d*^6^-DMSO): 13.67 (1H, s, NH of thione tautomer), 7.00 (1H, s, Ar-H), 5.97–5.84 (1H, m, CH), 5.21 [1H, dd, *J* = 1.2 + 10.4, N-CH_2_ (H_a_)], 5.04 [1H, dd, *J* = 1.2 + 17.2, N-CH_2_ (H_b_)], 4.73 (2H, d, *J* = 5.1, =CH_2_), 4.45 (2H, s, CH_2_), 2.34 (6H, s, 2CH_3_).

#### 4.5.5 5-(4,6-Dimethyl-pyrimidin-2-ylsulfanylmethyl)-4-(3-methoxy-propyl)-4*H*-[1,2,4]triazole-3-thiol (**8**)

To a solution of **4** (4.5 g, 21.2 mmol) in EtOH (170 mL) was added 3-methoxypropyl isothiocyanate (2.7 mL, 21.2 mmol) and the mixture was heated under reflux overnight. The reaction was cooled and concentrated under vacuum. To the yellow crystalline solid thus formed was added 1 M aq NaOH (25 mL) and the mixture was heated at 45 °C for 120 min before cooling. The reaction was neutralized with 10% aq HCl and was extracted with EtOAc. The extract was dried over Na_2_SO_4_, filtered, and concentrated. The resulting yellow solid was washed with Et_2_O and recrystallized from EtOH to afford **8** as an off-white crystalline solid (2.6 g, 38%). *m*/*z* (ES), found 324.0871 (C_13_H_18_N_5_OS_2_ [M−H]^−^) requires 324.1031; *δ*_H_/ppm (400 MHz, *d*^6^-DMSO): 13.60 (1H, s, NH of thione tautomer), 7.01 (1H, s, Ar-H), 4.51 (2H, s, CH_2_), 4.07 (2H, t, *J* = 7.3, *CH*_2_OCH_3_), 3.34 (2H, t, *J* = 6.0, N-CH_2_), 3.22 (3H, s, O*CH*_3_), 2.35 (6H, s, 2CH_3_), 2.16–1.92 (2H, m, CH_2_).

#### 4.5.6 5-(4,6-Dimethyl-pyrimidin-2-ylsulfanylmethyl)-4-(4-methoxy-phenyl)-4*H*-[1,2,4]triazole-3-thiol (**9**)

To a solution of **4** (4.5 g, 21.2 mmol) in EtOH (175 mL) was added 4-methoxyphenyl isothiocyanate (3.0 mL, 21.2 mmol) and the mixture was heated under reflux overnight. The reaction was cooled and concentrated under vacuum. To the yellow powder thus formed was added 1 M aq NaOH (50 mL) and the mixture was heated under reflux for 60 min before cooling. The reaction was neutralized with 10% aq HCl. The yellow precipitate formed was collected by filtration, was washed with EtOH–MeOH (1:1) and then vacuum dried to afford **9** as a pale yellow powder (5.9 g, 77%). *m*/*z* (ES), found 359.9088 (C_16_H_18_N_5_OS_2_ [M+H]^+^) requires 360.0875; *δ*_H_/ppm (400 MHz, *d*^6^-DMSO): 13.76 (1H, s, NH of thione tautomer), 7.30 (2H, d, *J* = 9.0, Ar-H), 6.99 (2H, d, *J* = 9.0, Ar-H), 6.93 (1H, s, Ar-H), 4.32 (2H, s, CH_2_), 3.78 (3H, s, OCH_3_), 2.28 (6H, s, 2CH_3_).

#### 4.5.7 4-(4-Chloro-phenyl)-5-(4,6-dimethyl-pyrimidin-2-ylsulfanylmethyl)-4*H*-[1,2,4]triazole-3-thiol (**10**)

To a solution of **4** (5.0 g, 23.6 mmol) in EtOH (175 mL) was added 4-chlorophenyl isothiocyanate (4.0 g, 23.6 mmol). The mixture was heated under reflux for 20 min and the formation of white precipitate was observed. The reaction was cooled and the white precipitate was collected by filtration and was washed with EtOH. To the precipitate was added 1 M aq NaOH (50 mL) and the mixture was heated at 45 °C for 45 min before cooling. The reaction was neutralized with 10% aq HCl. The white precipitate formed was collected by filtration, washed with MeOH and then vacuum dried to afford **10** as an off-white powder. In order to recover more **10** the filtrate was extracted with EtOAc. The extract was dried over Na_2_SO_4_, filtered, and concentrated. The resulting pale white powder was recrystallized from MeOH to afford **10** as a white powder. The combined yield of **10** was 4.9 g (57%). *m*/*z* (ES), found 363.8376 (C_15_H_15_ClN_5_S_2_ [M+H]^+^) requires 364.0379; *δ*_H_/ppm (400 MHz, *d*^6^-DMSO): 13.86 (1H, s, NH of thione tautomer), 7.52 (2H, d, *J* = 8.8, Ar-H), 7.43 (2H, d, *J* = 8.8, Ar-H), 6.93 (1H, s, Ar-H), 4.38 (2H, s, CH_2_), 2.28 (6H, s, 2CH_3_).

#### 4.5.8 2-Chloro-*N*-(3,4-dimethyl-phenyl)-acetamide (**13**)

To a solution of 3,4-dimethylaniline (1.85 g, 15 mmol) in CH_2_Cl_2_ (60 mL) was added Et_3_N (2.1 mL, 15.2 mmol) and the mixture was allowed to stir at room temperature (RT) for 10 min. To this was then added chloroacetyl chloride (1.19 mL, 15 mmol) and the reaction was left to stir at RT for 4 h. The reaction was concentrated under vacuum. The residue was diluted with H_2_O and extracted with EtOAc. The extract was washed with 1 M aq HCl, dried over Na_2_SO_4_, filtered, and concentrated under vacuum. The yellow precipitate formed was washed with Et_2_O and then vacuum dried. to afford **13** as a white powder (2.2 g, 74%). *m*/*z* (ES), found 198.0658 (C_10_H_13_ClNO [M+H]^+^) requires 198.0607; *δ*_H_/ppm (400 MHz, *d*^6^-DMSO): 10.11 (1H, s, NH), 7.35 (1H, s, Ar-H), 7.30 (1H, dd, *J* = 1.8 + 8.1, Ar-H), 7.07 (1H, d, *J* = 8.1, Ar-H), 4.21 (2H, s, CH_2_), 2.19 (3H, s, CH_3_), 2.16 (3H, s, CH_3_).

#### 4.5.9 2-Chloro-*N*-phenyl-acetamide (**14**)

Procedure as for **13** except using aniline (1.37 mL, 15 mmol) and the reaction mixture was allowed to stir at RT overnight. Pure **14** (without washing with Et_2_O) was obtained as off-white crystals (963 mg, 38%). *m*/*z* (ES), found 170.0979 (C_8_H_9_ClNO [M+H]^+^) requires 170.0294; *δ*_H_/ppm (400 MHz, *d*^6^-DMSO): 10.27 (1H, s, NH), 7.58 (2H, dd, *J* = 1.0 + 8.5, Ar-H), 7.36–7.30 (2H, m, Ar-H), 7.12–7.06 (1H, m, Ar-H), 4.25 (2H, s, CH_2_).

#### 4.5.10 2-Chloro-*N*-*m*-tolyl-acetamide (**15**)

Procedure as for **14** except using m-toluidine (1.62 mL, 15 mmol) and the reaction mixture was allowed to stir at RT overnight. Pure **15** was obtained as a brown powder (1.98 g, 72%). *m*/*z* (ES), found 184.0486 (C_9_H_11_ClNO [M+H]^+^) requires 184.0451; *δ*_H_/ppm (400 MHz, *d*^6^-DMSO): 10.19 (1H, s, NH), 7.42 (1H, s, Ar-H), 7.37 (1H, d, *J* = 8.4, Ar-H), 7.20 (1H, t, *J* = 7.8, Ar-H), 6.90 (1H, d, *J* = 7.5, Ar-H), 4.23 (2H, s, CH_2_), 2.28 (3H, s, CH_3_).

#### 4.5.11 2-Chloro-*N*-*p*-tolyl-acetamide (**16**)

Procedure as for **14** except using p-toluidine (1.61 g, 15 mmol) and the reaction mixture was allowed to stir at RT overnight. Pure **16** was obtained as a brown powder (1.81 g, 66%). *δ*_H_/ppm (400 MHz, *d*^6^-DMSO): 10.18 (1H, s, NH), 7.47 (2H, d, *J* = 8.4, Ar-H), 7.13 (2H, d, *J* = 8.2, Ar-H), 4.22 (2H, s, CH_2_), 2.25 (3H, s, CH_3_).

#### 4.5.12 2-Chloro-*N*-(2,5-dimethyl-phenyl)-acetamide (**17**)

Procedure as for **14** except using 2,5-dimethylaniline (1.87 mL, 15 mmol). The pale yellow precipitate formed was washed with PE–CH_2_Cl_2_ (4:1) and then vacuum dried. Pure **17** was obtained as a white powder (1.1 g, 37%). *m*/*z* (ES), found 198.1024 (C_10_H_13_ClNO [M+H]^+^) requires 198.0607; *δ*_H_/ppm (400 MHz, *d*^6^-DMSO): 9.57 (1H, s, NH), 7.20 (1H, s, Ar-H), 7.10 (1H, d, *J* = 7.7, Ar-H), 6.93 (1H, d, *J* = 7.6, Ar-H), 4.28 (2H, s, CH_2_), 2.25 (3H, s, CH_3_), 2.15 (3H, s, CH_3_).

#### 4.5.13 2-Chloro-*N*-(2,4-dimethyl-phenyl)-acetamide (**18**)

To a solution of 2,4-dimethylaniline (1.87 mL, 15 mmol) in CH_2_Cl_2_ (60 mL) was added Et_3_N (2.1 mL, 15 mmol) and the mixture was allowed to stir at RT for 10 min. To this was added chloroacetyl chloride (1.19 mL, 15 mmol) and the reaction was left to stir at RT for 4 h. The reaction was concentrated under vacuum. The residue was diluted with H_2_O and extracted with EtOAc. The extract was washed with 1 M aq HCl and was dried over MgSO_4_, filtered, and concentrated under vacuum. Pure **18** was obtained as a pale white powder (0.96 g, 32.5%). *m*/*z* (ES), found 198.1024 (C_10_H_13_ClNO [M+H]^+^) requires 198.0607; *δ*_H_/ppm (400 MHz, *d*^6^-DMSO): 9.59 (1H, s, NH), 7.24 (1H, d, *J* = 8.0, Ar-H), 7.03 (1H, s, Ar-H), 6.98 (1H, d, *J* = 8.1, Ar-H), 4.28 (2H, s, CH_2_), 2.25 (3H, s, CH_3_), 2.15 (3H, s, CH_3_).

#### 4.5.14 2-Chloro-*N*-(4-isopropyl-phenyl)-acetamide (**19**)

Procedure as for **14** except using 4-isopropylaniline (2.1 mL, 15 mmol) and the reaction mixture was allowed to stir at RT overnight. Pure **19** was obtained as a pale yellow powder (2.0 g, 63%). *m*/*z* (ES), found 212.0961 (C_11_H_15_ClNO [M+H]^+^) requires 212.0764; *δ*_H_/ppm (400 MHz, *d*^6^-DMSO): 10.19 (1H, s, NH), 7.49 (2H, d, *J* = 8.5, Ar-H), 7.19 (2H, d, *J* = 8.5, Ar-H), 4.22 (2H, s, CH_2_), 2.84 (1H, hept, CH of isopropyl), 1.18 [6H, d, *J* = 6.9, (CH_3_)_2_ of isopropyl].

#### 4.5.15 2-Chloro-*N*-(4-trifluoromethyl-phenyl)-acetamide (**20**)

Procedure as for **14** except using 4-trifluoromethylaniline (1.9 mL, 15 mmol). Pure **20** was obtained as a yellow powder (1.5 g, 42%). *m*/*z* (ES), found 235.6225 (C_9_H_6_ClF_3_NO [M−H]^−^) requires 236.0168; *δ*_H_/ppm (400 MHz, *d*^6^-DMSO): 10.64 (1H, s, NH), 7.80 (2H, d, *J* = 8.5, Ar-H), 7.70 (2H, d, *J* = 8.6, Ar-H), 4.30 (2H, s, CH_2_).

#### 4.5.16 *N*-(3,5-Bis-trifluoromethyl-phenyl)-2-chloro-acetamide (**21**)

Procedure as for **14** except using 3,5-bis(trifluoromethyl)aniline (2.4 mL, 15 mmol) and the mixture was allowed to stir at RT overnight. The precipitate obtained was washed with PE and vacuum dried. Pure **21** was obtained as a brick-red powder (1.77 g, 39%). *δ*_H_/ppm (400 MHz, *d*^6^-DMSO): 10.93 (1H, s, NH), 8.25 (2H, s, Ar-H), 7.80 (1H, s, Ar-H), 4.34 (2H, s, CH_2_).

#### 4.5.17 2-Chloro-*N*-(4-methoxy-phenyl)-acetamide (**22**)

Procedure as for **14** except using *p*-anisidine (1.85 g, 15 mmol). Pure **22** was obtained as a gray powder (1.77 g, 59%). *m*/*z* (ES), found 200.0450 (C_9_H_11_ClNO_2_ [M+H]^+^) requires 200.0400; *δ*_H_/ppm (400 MHz, *d*^6^-DMSO): 10.13 (1H, s, NH), 7.50 (2H, d, *J* = 9.1, Ar-H), 6.90 (2H, d, *J* = 9.1, Ar-H), 4.21 (2H, s, CH_2_), 3.72 (3H, s, OCH_3_).

#### 4.5.18 2-Chloro-*N*-(4-chloro-phenyl)-acetamide (**23**)

Procedure as for **14** except using 4-chloroaniline (1.95 g, 15 mmol). Pure **23** was obtained as a pale yellow powder (2.26 g, 74%). *m*/*z* (ES), found 201.6550 (C_8_H_6_Cl_2_NO [M−H]^−^) requires 201.9905; *δ*_H_/ppm (400 MHz, *d*^6^-DMSO): 10.41 (1H, s, NH), 7.62 (2H, d, *J* = 9.0, Ar-H), 7.38 (2H, d, *J* = 8.9, Ar-H), 4.25 (2H, s, CH_2_).

#### 4.5.19 *N*-(4-Bromo-phenyl)-2-chloro-acetamide (**24**)

To a solution of 4-bromoaniline (2.66 g, 15 mmol) in CH_2_Cl_2_ (60 mL) was added Et_3_N (2.1 mL, 15.2 mmol) and the mixture was allowed to stir at RT for 10 min. To this was added chloroacetyl chloride (1.19 mL, 15 mmol) and the reaction was left to stir at RT for 4 h. The white precipitate formed was separated by filtration and washed with CH_2_Cl_2_. Pure **24** was obtained as a white powder (898 mg, 24%). *m*/*z* (ES), found 247.9191 (C_8_H_8_BrClNO [M+H]^+^) requires 247.9400; *δ*_H_/ppm (400 MHz, *d*^6^-DMSO): 10.41 (1H, s, NH), 7.56 (2H, d, *J* = 9.0, Ar-H), 7.51 (2H, d, *J* = 9.0, Ar-H), 4.25 (2H, s, CH_2_).

#### 4.5.20 2-Chloro-*N*-thiazol-2-yl-acetamide (**25**)

Procedure as for **14** except using 2-aminothiazole (1.55 g, 15 mmol). Precipitation was observed within 15 min of the addition of chloroacetyl chloride. The precipitate was extracted with EtOAc. Pure **25** was obtained as a pale yellow powder (642 mg, 24%) after recrystallization from EtOH. *m*/*z* (ES), found 176.9838 (C_5_H_6_ClN_2_OS [M+H]^+^) requires 176.9811; *δ*_H_/ppm (400 MHz, *d*^6^-DMSO): 12.48 (1H, s, NH), 7.50 (1H, d, *J* = 3.6, Ar-H), 7.27 (1H, d, *J* = 3.6, Ar-H), 4.38 (2H, s, CH_2_).

#### 4.5.21 2-Chloro-*N*-(4-methyl-thiazol-2-yl)-acetamide (**25a**)

Procedure as for **14** except using 2-amino-4-methylthiazole (1.75 g, 15 mmol). Pure **25a** was obtained as a pale yellow powder (1.4 g, 49%). *m*/*z* (ES), found 190.0078 (C_6_H_8_ClN_2_OS [M+H]^+^) requires 190.9968; *δ*_H_/ppm (400 MHz, *d*^6^-DMSO): 12.42 (1H, s, NH), 6.80 (1H, d, *J* = 1.0, Ar-H), 4.35 (2H, s, CH_2_), 2.26 (3H, d, *J* = 1.0, Ar-H).

#### 4.5.22 2-Chloro-*N*-(5-methyl-isoxazol-3-yl)-acetamide (**25b**)

Procedure as for **14** except using 3-amino-5-methyl-isoxazole (1.5 g, 15 mmol, 1.00 equiv) and the reaction mixture was allowed to stir at RT overnight. The precipitate obtained was washed with CH_2_Cl_2_ and then vacuum dried. Pure **25b** was obtained as a white crystalline solid (766 mg, 30%). *m*/*z* (ES), found 175.0221 (C_6_H_8_ClN_2_O_2_ [M+H]^+^) requires 175.0196; *δ*_H_/ppm (400 MHz, *d*^6^-DMSO): 11.25 (1H, s, NH), 6.62 (1H, s, Ar-H), 4.29 (2H, s, CH_2_), 2.38 (3H, s, CH_3_).

#### 4.5.23 3-Chloro-*N*-(3,4-dimethyl-phenyl)-propionamide (**26**)

Procedure as for **14** except using 3-chloropropionyl chloride (1.44 mL, 15 mmol) and the reaction mixture was allowed to stir at RT overnight. The precipitate obtained was washed with PE and then vacuum dried. Pure **26** was obtained as a white powder (1.9 g, 60%). *m*/*z* (ES), found 212.1006 (C_11_H_15_ClNO [M+H]^+^) requires 212.0764; *δ*_H_/ppm (400 MHz, *d*^6^-DMSO): 9.87 (1H, s, NH), 7.37 (1H, s, Ar-H), 7.31 (1H, dd, *J* = 2.1 + 8.1, Ar-H), 7.04 (1H, d, *J* = 8.1, Ar-H), 3.86 (2H, t, *J* = 6.3, CH_2_), 2.78 (2H, t, *J* = 6.3, CH_2_), 2.19 (3H, s, CH_3_), 2.16 (3H, s, CH_3_).

#### 4.5.31 *N*-(2,5-Dimethyl-phenyl)-2-[5-(4,6-dimethyl-pyrimidin-2-ylsulfanylmethyl)-4-furan-2-ylmethyl-4*H*-[1,2,4]triazol-3-ylsulfanyl]-acetamide (**1a**)

A solution of **6** (333 mg, 1 mmol) in DMF (3 mL), and a solution of **17** (198 mg, 1 mmol) in DMF (2 mL) were combined in a dry Radleys reaction tube containing K_2_CO_3_ (166 mg, 1.2 mmol). The mixture was allowed to stir at 40 °C overnight in a Radleys parallel synthesizer. The mixture was poured on crushed ice (75 mL) and allowed to stand for a few hours. The mixture was then extracted with EtOAc. The combined extracts were washed with distilled H_2_O, dried over Na_2_SO_4_, filtered, and concentrated under vacuum. Purification of the crude product by flash column chromatography (FCC; EtOAc, *R_f_* = 0.26) gave pure **1a** as a pale white powder (196 mg, 40%). *δ*_H_/ppm (400 MHz, *d*^6^-DMSO): 9.57 (1H, s, NH), 7.63 (1H, dd, *J* = 0.7 + 1.7, Ar-H), 7.22 (1H, s, Ar-H), 7.05 (1H, d, *J* = 7.7, Ar-H), 6.97 (1H, s, Ar-H), 6.88 (1H, d, *J* = 7.7, Ar-H), 6.48–6.42 (2H, m, Ar-H), 5.39 (2H, s, CH_2_), 4.65 (2H, s, CH_2_), 4.11 (2H, s, CH_2_), 2.33 [6H, s, (CH_3_)_2_], 2.23 (3H, s, CH_3_), 2.06 (3H, s, CH_3_).

#### 4.5.32 2-[5-(4,6-Dimethyl-pyrimidin-2-ylsulfanylmethyl)-4-furan-2-ylmethyl-4*H*-[1,2,4]triazol-3-ylsulfanyl]-acetamide (**27**)

Procedure as for **1a** except using 2-bromoacetamide (138 mg, 1 mmol) and the reaction was carried out for 48 h. The white precipitate formed was collected by filtration, washed with H_2_O and lyophilized, followed by recrystallization from EtOH. Pure **27** was obtained as a pale white powder (173 mg, 45%). *m*/*z* (ES), found 389.0885 (C_16_H_17_N_6_O_2_S_2_ [M−H]^−^) requires 389.0933; *δ*_H_/ppm (400 MHz, *d*^6^-DMSO): 7.57–7.70 (2H, m, Ar-H), 7.20 (1H, br s, OH), 6.99 (1H, s, =NH), 6.40–6.50 (2H, m, Ar-H), 5.36 (2H, s, CH_2_), 4.64 (2H, s, CH_2_), 3.85 (2H, s, CH_2_), 2.34 (6H, s, 2CH_3_).

#### 4.5.33 2-[5-(4,6-Dimethyl-pyrimidin-2-ylsulfanylmethyl)-4-furan-2-ylmethyl-4*H*-[1,2,4]triazol-3-ylsulfanyl]-*N*-thiazol-2-yl-acetamide (**28**)

Procedure as for **1a** except using **25** (177 mg, 1 mmol) and the reaction was carried out for 24 h. The precipitate formed was collected by filtration, washed thoroughly with water and lyophilized, followed by recrystallization from MeOH. Pure **28** (EtOAc, *R_f_* = 0.06) was obtained as a white powder (292 mg, 62%). *m*/*z* (ES), found 474.6843 (C_19_H_20_N_7_O_2_S_3_ [M+H]^+^) requires 474.0762; *δ*_H_/ppm (400 MHz, *d*^6^-DMSO): 12.38 (1H, s, NH), 7.63 (1H, dd, *J* = 0.8 + 1.8, Ar-H), 7.47 (1H, d, *J* = 3.6, Ar-H), 7.22 (1H, d, *J* = 3.6, Ar-H), 6.98 (1H, s, Ar-H), 6.52–6.40 (2H, m, Ar-H), 5.38 (2H, s, CH_2_), 4.63 (2H, s, CH_2_), 4.19 (2H, s, CH_2_), 2.34 (6H, s, 2CH_3_).

#### 4.5.34 2-[5-(4,6-Dimethyl-pyrimidin-2-ylsulfanylmethyl)-4-furan-2-ylmethyl-4*H*-[1,2,4]triazol-3-ylsulfanyl]-*N*-(4-methyl-thiazol-2-yl)-acetamide (**29**)

Procedure as for **1a** except using **25a** (191 mg, 1 mmol) and the reaction was carried out for 48 h. The mixture was extracted with EtOAc. The combined extracts were washed with H_2_O and dried over Na_2_SO_4_, filtered, and concentrated under vacuum. Purification of the crude product by FCC (EtOAc–MeOH (95:5) gave **29** as a pale white powder (219 mg, 45%). *m*/*z* (ES), found 486.0944 (C_20_H_20_N_7_O_2_S_3_ [M−H]^−^) requires 486.0919; *δ*_H_/ppm (400 MHz, *d*^6^-DMSO): 12.29 (1H, s, NH), 7.63 (1H, dd, *J* = 0.8 + 1.8, Ar-H), 6.98 (1H, s, Ar-H), 6.76 (1H, d, *J* = 1.0, Ar-H), 6.50–6.40 (2H, m, Ar-H), 5.37 (2H, s, CH_2_), 4.63 (2H, s, CH_2_), 4.16 (2H, s, CH_2_), 2.34 (6H, s, 2CH_3_), 2.25 (3H, d, *J* = 0.9, CH_3_).

#### 4.5.35 2-[5-(4,6-Dimethyl-pyrimidin-2-ylsulfanylmethyl)-4-furan-2-ylmethyl-4*H*-[1,2,4]triazol-3-ylsulfanyl]-*N*-(5-methyl-isoxazol-3-yl)-acetamide (**30**)

Procedure as for **1a** except using **25b** (175 mg, 1 mmol) and the reaction was carried out for 48 h. The precipitate was collected by filtration, washed with H_2_O and lyophilized, followed by recrystallization from MeOH. Pure **30** was obtained as a white powder (298 mg, 65%). *m*/*z* (ES), found 472.1485 (C_20_H_22_N_7_O_3_S_2_ [M+H]^+^) requires 472.1147; *δ*_H_/ppm (400 MHz, *d*^6^-DMSO): 11.20 (1H, s, NH), 7.63 (1H, dd, *J* = 0.8 + 1.8, Ar-H), 6.99 (1H, s, Ar-H), 6.56 (1H, s, Ar-H), 6.48–6.42 (2H, m, Ar-H), 5.37 (2H, s, CH_2_), 4.63 (2H, s, CH_2_), 4.11 (2H, s, CH_2_), 2.36 (3H, d, *J* = 0.8, CH_3_), 2.34 (6H, s, 2CH_3_).

#### 4.5.36 2-[5-(4,6-Dimethyl-pyrimidin-2-ylsulfanylmethyl)-4-furan-2-ylmethyl-4*H*-[1,2,4]triazol-3-ylsulfanyl]-*N*-phenyl-acetamide (**31**)

Procedure as for **1a** except using **6** (600 mg, 1.8 mmol) and **14** (305 mg, 1.8 mmol). The mixture was extracted with EtOAc. The combined extracts were washed with H_2_O and dried over Na_2_SO_4_, filtered, and concentrated under vacuum. Purification of the crude product by FCC (EtOAc, *R_f_* = 0.20), gave **31** as a pale white powder (586 mg, 70%). *m*/*z* (ES), found 465.1360 (C_22_H_21_N_6_O_2_S_2_ [M−H]^−^) requires 465.1246; *δ*_H_/ppm (400 MHz, *d*^6^-DMSO): 10.28 (1H, s, NH), 7.63 (1H, dd, *J* = 0.8 + 1.8, Ar-H), 7.50–7.56 (2H, m, Ar-H), 7.26–7.32 (2H, m, Ar-H), 7.05 (1H, t, *J* = 7.4, Ar-H), 6.98 (1H, s, Ar-H), 6.42–6.49 (2H, m, Ar-H), 5.39 (2H, s, CH_2_), 4.64 (2H, s, CH_2_), 4.11 (2H, s, CH_2_), 2.34 (6H, s, 2CH_3_).

#### 4.5.37 2-[5-(4,6-Dimethyl-pyrimidin-2-ylsulfanylmethyl)-4-furan-2-ylmethyl-4*H*-[1,2,4]triazol-3-ylsulfanyl]-*N*-*m*-tolyl-acetamide (**32**)

Procedure as for **1a** except using **15** (184 mg, 1 mmol) and the reaction was carried out for 24 h. The mixture was extracted with EtOAc. The combined extracts were washed with H_2_O and dried over Na_2_SO_4_, filtered, and concentrated under vacuum. Purification of the crude product by FCC (EtOAc, *R_f_* = 0.21), gave **32** as a pale white powder (264 mg, 55%). *m*/*z* (ES), found 479.1382 (C_23_H_23_N_6_O_2_S_2_ [M−H]^−^) requires 479.1402; *δ*_H_/ppm (400 MHz, *d*^6^-DMSO): 10.20 (1H, s, NH), 7.63 (1H, dd, *J* = 0.8 + 1.8, Ar-H), 7.37 (1H, s, Ar-H), 7.31 (1H, d, *J* = 8.3, Ar-H), 7.17 (1H, t, *J* = 7.7/7.9, Ar-H), 6.98 (1H, s, Ar-H), 6.87 (1H, d, *J* = 7.5, Ar-H), 6.42–6.49 (2H, m, Ar-H), 5.39 (2H, s, CH_2_), 4.64 (2H, s, CH_2_), 4.09 (2H, s, CH_2_), 2.34 (6H, s, 2CH_3_), 2.27 (3H, s, CH_3_).

#### 4.5.38 2-[5-(4,6-Dimethyl-pyrimidin-2-ylsulfanylmethyl)-4-furan-2-ylmethyl-4*H*-[1,2,4]triazol-3-ylsulfanyl]-*N*-*p*-tolyl-acetamide (**33**)

Procedure as for **1a** except using **16** (184 mg, 1 mmol). The mixture was extracted with EtOAc. The combined extracts were washed with H_2_O and dried over Na_2_SO_4_, filtered, and concentrated under vacuum followed by recrystallization from MeOH. Pure **33** was obtained as a white crystalline solid (195 mg, 40%). *m*/*z* (ES), found 479.1350 (C_23_H_23_N_6_O_2_S_2_ [M−H]^−^) requires 479.1402; *δ*_H_/ppm (400 MHz, *d*^6^-DMSO): 10.19 (1H, s, NH), 7.63 (1H, dd, *J* = 0.8 + 1.8, Ar-H), 7.41 (2H, d, *J* = 8.5, Ar-H), 7.09 (2H, d, *J* = 8.3, Ar-H), 6.98 (1H, s, Ar-H), 6.41–6.49 (2H, m, Ar-H), 5.39 (2H, s, CH_2_), 4.64 (2H, s, CH_2_), 4.08 (2H, s, CH_2_), 2.34 (6H, s, 2CH_3_), 2.24 (3H, s, CH_3_).

#### 4.5.39 *N*-(2,4-Dimethyl-phenyl)-2-[5-(4,6-dimethyl-pyrimidin-2-ylsulfanylmethyl)-4-furan-2-ylmethyl-4*H*-[1,2,4]triazol-3-ylsulfanyl]-acetamide (**34**)

Procedure as for **1a** except using **6** (200 mg, 0.6 mmol), **18** (118 mg, 0.6 mmol) and the reaction was carried out for 48 h. The mixture was extracted with EtOAc. The combined extracts were washed with H_2_O and dried over Na_2_SO_4_, filtered, and concentrated under vacuum. Purification of the crude product by FCC (EtOAc, *R_f_* = 0.23), gave **34** as a pale white powder (147 mg, 50%). *m*/*z* (ES), found 493.1446 (C_24_H_25_N_6_O_2_S_2_ [M−H]^−^) requires 493.1559; *δ*_H_/ppm (400 MHz, *d*^6^-DMSO): 9.55 (1H, s, NH), 7.65–7.62 (1H, m, Ar-H), 7.23 (1H, d, *J* = 8.0, Ar-H), 7.01–6.90 (3H, m, Ar-H), 6.48–6.42 (2H, m, Ar-H), 5.39 (2H, s, CH_2_), 4.65 (2H, s, CH_2_), 4.09 (2H, s, CH_2_), 2.33 (6H, s, 2CH_3_), 2.23 (3H, s, CH_3_), 2.06 (3H, s, CH_3_).

#### 4.5.40 *N*-(3,4-Dimethyl-phenyl)-2-[5-(4,6-dimethyl-pyrimidin-2-ylsulfanylmethyl)-4-furan-2-ylmethyl-4*H*-[1,2,4]triazol-3-ylsulfanyl]-acetamide (**35**)

A solution of **6** (1.0 g, 3 mmol) in DMF (8 mL) and a solution of **13** (593 mg, 3 mmol) in DMF (4 mL) were combined in a dry Radleys reaction tube containing K_2_CO_3_ (442 mg, 3.2 mmol). The mixture was allowed to stir at 40 °C overnight in a Radleys parallel synthesizer. The mixture was poured on crushed ice (50 mL) and allowed to stand for few hours. The brown precipitate formed was collected by filtration, washed with H_2_O and lyophilized, followed by recrystallization from EtOH–MeOH (1:1). Pure **35** was obtained as a pale yellow crystalline solid (952 mg, 64%). *m*/*z* (ES), found 495.1811 (C_24_H_27_N_6_O_2_S_2_ [M+H]^+^) requires 495.1559; *δ*_H_/ppm (400 MHz, *d*^6^-DMSO): 10.14 (1H, s, NH), 7.64 (1H, dd, *J* = 0.8 + 1.8, Ar-H), 7.31 (1H, d, *J* = 1.8, Ar-H), 7.24 (1H, dd, *J* = 2.1 + 8.1, Ar-H), 7.03 (1H, d, *J* = 8.2, Ar-H), 6.98 (1H, s, Ar-H), 6.50–6.41 (2H, m, Ar-H), 5.39 (2H, s, CH_2_), 4.64 (2H, s, CH_2_), 4.07 (2H, s, CH_2_), 2.33 (6H, s, 2CH_3_), 2.17 (3H, s, CH_3_), 2.15 (3H, s, CH_3_).

#### 4.5.41 2-[5-(4,6-Dimethyl-pyrimidin-2-ylsulfanylmethyl)-4-furan-2-ylmethyl-4*H*-[1,2,4]triazol-3-ylsulfanyl]-*N*-(4-isopropyl-phenyl)-acetamide (**36**)

Procedure as for **1a** except using **19** (211 mg, 1 mmol). The mixture was extracted with EtOAc. The combined extracts were washed with H_2_O and dried over Na_2_SO_4_, filtered, and concentrated under vacuum. Purification of the crude product by FCC (EtOAc, *R_f_* = 0.21), gave **36** as a pale white powder (300 mg, 59%). *m*/*z* (ES), found 509.7175 (C_25_H_29_N_6_O_2_S_2_ [M+H]^+^) requires 509.1715; *δ*_H_/ppm (400 MHz, *d*^6^-DMSO): 10.20 (1H, s, NH), 7.63 (1H, dd, *J* = 0.8 + 1.8, Ar-H), 7.43 (2H, d, *J* = 8.6, Ar-H), 7.16 (2H, d, *J* = 8.5, Ar-H), 6.98 (1H, s, Ar-H), 6.48–6.42 (2H, m, Ar-H), 5.39 (2H, s, CH_2_), 4.64 (2H, s, CH_2_), 4.08 (2H, s, CH_2_), 2.83 (1H, hept, CH of isopropyl), 2.34 (6H, s, 2CH_3_), 1.17 [6H, d, *J* = 6.9, (CH_3_)_2_].

#### 4.5.42 2-[5-(4,6-Dimethyl-pyrimidin-2-ylsulfanylmethyl)-4-furan-2-ylmethyl-4*H*-[1,2,4]triazol-3-ylsulfanyl]-*N*-(4-trifluoromethyl-phenyl)-acetamide (**37**)

Procedure as for **1a** except using **20** (238 mg, 1 mmol). The precipitate was collected by filtration, washed with H_2_O and lyophilized, followed by recrystallization from MeOH. Pure **37** was obtained as a white powder (314 mg, 59%). *m*/*z* (ES), found 535.6185 (C_23_H_22_F_3_N_6_O_2_S_2_ [M+H]^+^) requires 535.1119; *δ*_H_/ppm (400 MHz, *d*^6^-DMSO): 10.65 (1H, s, NH), 7.74 (2H, d, *J* = 8.6, Ar-H), 7.67 (2H, d, *J* = 8.7, Ar-H), 7.63 (1H, dd, *J* = 0.8 + 1.8, Ar-H), 6.98 (1H, s, Ar-H), 6.48–6.42 (2H, m, Ar-H), 5.39 (2H, s, CH_2_), 4.64 (2H, s, CH_2_), 4.15 (2H, s, CH_2_), 2.33 (6H, s, 2CH_3_).

#### 4.5.43 *N*-(3,5-Bis-trifluoromethyl-phenyl)-2-[5-(4,6-dimethyl-pyrimidin-2-ylsulfanylmethyl)-4-furan-2-ylmethyl-4*H*-[1,2,4]triazol-3-ylsulfanyl]-acetamide (**38**)

Procedure as for **1a** except using **21** (306 mg, 1 mmol) and the reaction was carried out for 48 h. The precipitate was collected by filtration, washed with H_2_O and lyophilized, followed by recrystallization from EtOH. Pure **38** was obtained as a white powder (264 mg, 44%). *m*/*z* (ES), found 603.9979 (C_24_H_21_F_6_N_6_O_2_S_2_ [M+H]^+^) requires 603.0993; *δ*_H_/ppm (400 MHz, *d*^6^-DMSO): 10.95 (1H, s, NH), 8.20 (2H, s, Ar-H), 7.78 (1H, s, Ar-H), 7.63 (1H, dd, *J* = 0.8 + 1.8, Ar-H), 6.96 (1H, s, Ar-H), 6.48–6.42 (2H, m, Ar-H), 5.39 (2H, s, CH_2_), 4.63 (2H, s, CH_2_), 4.16 (2H, s, CH_2_), 2.33 (6H, s, 2CH_3_).

#### 4.5.44 *N*-(4-Chloro-phenyl)-2-[5-(4,6-dimethyl-pyrimidin-2-ylsulfanylmethyl)-4-furan-2-ylmethyl-4*H*-[1,2,4]triazol-3-ylsulfanyl]-acetamide (**39**)

Procedure as for **1a** except using **23** (204 mg, 1 mmol) and the reaction was carried out for 24 h. The precipitate was collected by filtration, washed with H_2_O and lyophilized, followed by recrystallization from EtOH–MeOH (1:1). Pure **39** was obtained as a white powder (227 mg, 46%). *m*/*z* (ES), found 500.6534 (C_22_H_22_ClN_6_O_2_S_2_ [M+H]^+^) requires 501.0856; *δ*_H_/ppm (400 MHz, *d*^6^-DMSO): 10.42 (1H, s, NH), 7.63 (1H, dd, *J* = 0.8 + 1.8, Ar-H), 7.56 (2H, d, *J* = 8.9, Ar-H), 7.35 (2H, d, *J* = 8.9, Ar-H), 6.98 (1H, s, Ar-H), 6.49–6.42 (2H, m, Ar-H), 5.39 (2H, s, CH_2_), 4.64 (2H, s, CH_2_), 4.10 (2H, s, CH_2_), 2.33 (6H, s, 2CH_3_).

#### 4.5.45 *N*-(4-Bromo-phenyl)-2-[5-(4,6-dimethyl-pyrimidin-2-ylsulfanylmethyl)-4-furan-2-ylmethyl-4*H*-[1,2,4]triazol-3-ylsulfanyl]-acetamide (**40**)

Procedure as for **1a** except using **6** (200 mg, 0.6 mmol), **24** (149 mg, 0.6 mmol) and the reaction was carried out for 36 h. The precipitate was collected by filtration, washed with H_2_O and lyophilized, followed by recrystallization from MeOH. Pure **40** was obtained as a white powder (185 mg, 57%). *m*/*z* (ES), found 544.9952 (C_22_H_22_BrN_6_O_2_S_2_ [M+H]^+^) requires 545.0351; *δ*_H_/ppm (400 MHz, *d*^6^-DMSO): 10.41 (1H, s, NH), 7.63 (1H, dd, *J* = 0.8 + 1.8, Ar-H), 7.54–7.45 (4H, m, Ar-H), 6.98 (1H, s, Ar-H), 6.48–6.42 (2H, m, Ar-H), 5.39 (2H, s, CH_2_), 4.63 (2H, s, CH_2_), 4.10 (2H, s, CH_2_), 2.34 (6H, s, 2CH_3_).

#### 4.5.46 2-[4-Allyl-5-(4,6-dimethyl-pyrimidin-2-ylsulfanylmethyl)-4*H*-[1,2,4]triazol-3-ylsulfanyl]-*N*-(3,4-dimethyl-phenyl)-acetamide (**41**)

A solution of **7** (293 mg, 1 mmol) in DMF (3 mL) and a solution of **13** (198 mg, 1 mmol) in DMF (2 mL) were combined in a dry Radleys reaction tube containing K_2_CO_3_ (166 mg, 1.2 mmol). The mixture was allowed to stir at 40 °C overnight in a Radleys parallel synthesizer. The mixture was poured on crushed ice (50 mL) and allowed to stand for few hours. The brown precipitate formed was collected by filtration, washed with H_2_O and lyophilized, followed by recrystallization from EtOH. Pure **41** was obtained as pale white crystals (96 mg, 21%). *m*/*z* (ES), found 453.1533 (C_22_H_25_N_6_OS_2_ [M−H]^−^) requires 453.1610; *δ*_H_/ppm (400 MHz, *d*^6^-DMSO): 10.10 (1H, s, NH), 7.30 (1H, d, *J* = 1.8, Ar-H), 7.24 (1H, dd, *J* = 2.1 + 8.1, Ar-H), 7.04 (1H, d, *J* = 8.2, Ar-H), 7.00 (1H, s, Ar-H), 6.04–5.83 (1H, m, CH of allyl), 5.20 [1H, dd, *J* = 1.0 + 10.4, N-CH_2_ (H_a_)], 4.91 [1H, dd, *J* = 1.0 + 17.2, N-CH_2_ (H_b_)], 4.73 (2H, d, *J* = 5.0, =CH_2_), 4.55 (2H, s, CH_2_), 4.05 (2H, s, CH_2_), 2.35 (6H, s, 2CH_3_), 2.17 (3H, s, CH_3_), 2.15 (3H, s, CH_3_).

#### 4.5.47 *N*-(3,4-Dimethyl-phenyl)-2-[5-(4,6-dimethyl-pyrimidin-2-ylsulfanylmethyl)-4-(3-methoxy-propyl)-4*H*-[1,2,4]triazol-3-ylsulfanyl]-acetamide (**42**)

A solution of **8** (325 mg, 1 mmol) in DMF (3 mL) and a solution of **13** (198 mg, 1 mmol) in DMF (2 mL) were combined in a dry Radleys reaction tube containing K_2_CO_3_ (166 mg, 1.2 mmol). The mixture was allowed to stir at 40 °C overnight in a Radleys parallel synthesizer. The mixture was poured on crushed ice (50 mL) and allowed to stand for few hours. The precipitate formed was collected by filtration, washed with H_2_O and lyophilized, followed by recrystallization from EtOH. Pure **42** was obtained as a crystalline white solid (317 mg, 65%). *m*/*z* (ES), found 487.1689 (C_23_H_31_N_6_O_2_S_2_ [M+H]^+^) requires 487.1872; *δ*_H_/ppm (400 MHz, *d*^6^-DMSO): 10.11 (1H, s, NH), 7.30 (1H, s, Ar-H), 7.24 (1H, d, *J* = 8.1, Ar-H), 7.04 (1H, d, *J* = 8.0, Ar-H), 7.00 (1H, s, Ar-H), 4.58 (2H, s, CH_2_), 4.10 (2H, t, *J* = 7.2, *CH*_2_OCH_3_), 4.08 (2H, s, CH_2_), 3.29 (2H, t, *J* = 5.8, N-CH_2_), 3.21 (3H, d, *J* = 1.7, O*CH*_3_), 2.35 (6H, s, 2CH_3_), 2.17 (3H, s, CH_3_), 2.15 (3H, s, CH_3_), 1.98–1.86 (2H, m, CH_2_).

#### 4.5.48 *N*-(3,4-Dimethyl-phenyl)-2-[5-(4,6-dimethyl-pyrimidin-2-ylsulfanylmethyl)-4-(4-methoxy-phenyl)-4*H*-[1,2,4]triazol-3-ylsulfanyl]-acetamide (**43**)

A solution of **9** (360 mg, 1 mmol, 1 equiv) in DMF (4 mL) and a solution of **13** (198 mg, 1 mmol) in DMF (2 mL) were combined in a dry Radleys reaction tube containing K_2_CO_3_ (166 mg, 1.2 mmol). The mixture was allowed to stir at 40 °C for 36 h in a Radleys parallel synthesizer. The mixture was poured on crushed ice (25 mL) and allowed to stand for few hours. The precipitate formed was collected by filtration, washed with H_2_O and lyophilized. Pure **43** was obtained as a pale yellow powder (469 mg, 90%). *m*/*z* (ES), found 519.1627 (C_26_H_27_N_6_O_2_S_2_ [M−H]^−^) requires 519.1715; *δ*_H_/ppm (400 MHz, *d*^6^-DMSO): 10.13 (1H, s, NH), 7.35 (2H, d, *J* = 8.9, Ar-H), 7.32–7.23 (2H, m, Ar-H), 7.04 (1H, d, *J* = 8.2, Ar-H), 6.99 (2H, d, *J* = 8.9, Ar-H), 6.91 (1H, s, Ar-H), 4.46 (2H, s, CH_2_), 4.08 (2H, s, CH_2_), 3.78 (3H, s, OCH_3_), 2.27 (6H, s, 2CH_3_), 2.17 (3H, s, CH_3_), 2.16 (3H, s, CH_3_).

#### 4.5.49 2-[4-(4-Chloro-phenyl)-5-(4,6-dimethyl-pyrimidin-2-ylsulfanylmethyl)-4*H*-[1,2,4]triazol-3-ylsulfanyl]-*N*-(3,4-dimethyl-phenyl)-acetamide (**44**)

A solution of **10** (364 mg, 1 mmol) in DMF (3 mL) and a solution of **13** (198 mg, 1 mmol) in DMF (2 mL) were combined in a dry Radleys reaction tube containing K_2_CO_3_ (166 mg, 1.2 mmol). The mixture was allowed to stir at 40 °C overnight in a Radleys parallel synthesizer. The mixture was poured on crushed ice (50 mL) and allowed to stand for few hours. The brown precipitate formed was collected by filtration, washed with H_2_O and lyophilized. Without any further purification, **44** was obtained as a white powder (481 mg, 92%). *m*/*z* (ES), found 523.1134 (C_25_H_24_ClN_6_OS_2_ [M−H]^−^) requires 523.1220; *δ*_H_/ppm (400 MHz, *d*^6^-DMSO): 10.11 (1H, s, NH), 7.55–7.46 (4H, m Ar-H), 7.33–7.22 (2H, m, Ar-H), 7.04 (1H, d, *J* = 8.14, Ar-H), 6.92 (1H, s, Ar-H), 4.51 (2H, s, CH_2_), 4.08 (2H, s, CH_2_), 2.27 (6H, s, 2CH_3_), 2.18 (3H, s, CH_3_), 2.16 (3H, s, CH_3_).

#### 4.5.50 *N*-(3,4-Dimethyl-phenyl)-3-[5-(4,6-dimethyl-pyrimidin-2-ylsulfanylmethyl)-4-furan-2-ylmethyl-4*H*-[1,2,4]triazol-3-ylsulfanyl]-propionamide (**45**)

Procedure as for **1a** except using **26** (212 mg, 1 mmol) and the reaction was carried out for 36 h. The mixture was extracted with EtOAc. The combined extracts were washed with H_2_O and dried over Na_2_SO_4_, filtered, and concentrated under vacuum. Purification of the crude product by FCC (EtOAc, *R_f_* = 0.12), gave **45** as a pale white powder (127 mg, 25%). *m*/*z* (ES), found 509.1672 (C_25_H_29_N_6_O_2_S_2_ [M+H]^+^) requires 509.1715; *δ*_H_/ppm (400 MHz, *d*^6^-DMSO): 9.83 (1H, s, NH), 7.60 (1H, dd, *J* = 0.9 + 1.7, Ar-H), 7.35 (1H, d, *J* = 1.8, Ar-H), 7.28 (1H, dd, *J* = 2.1 + 8.1, Ar-H), 7.03 (1H, d, *J* = 8.2, Ar-H), 7.00 (1H, s, Ar-H), 6.43–6.37 (2H, m, Ar-H), 5.30 (2H, s, CH_2_), 4.64 (2H, s, CH_2_), 3.34 (2H, t, *J* = 6.8, CH_2_), 2.76 (2H, t, *J* = 6.7/6.8, CH_2_), 2.34 (6H, s, 2CH_3_), 2.17 (3H, s, CH_3_), 2.15 (3H, s, CH_3_).

#### 4.5.51 2-[4-Allyl-5-(4,6-dimethyl-pyrimidin-2-ylsulfanylmethyl)-4*H*-[1,2,4]triazol-3-ylsulfanyl]-*N*-(4-isopropyl-phenyl)-acetamide (**46**)

Procedure as for **1a** except using **19** (212 mg, 1 mmol) and the reaction was carried out for 22 h. The mixture was extracted with EtOAc. The combined extracts were washed H_2_O and dried over Na_2_SO_4_, filtered, and concentrated under vacuum. Purification of the crude product by FCC (EtOAc, *R_f_* = 0.16), gave **46** as a pale white powder (228 mg, 49%). *m*/*z* (ES), found 469.2179 (C_23_H_29_N_6_OS_2_ [M+H]^+^) requires 469.1766; *δ*_H_/ppm (400 MHz, *d*^6^-DMSO): 10.19 (1H, s, NH), 7.43 (2H, d, *J* = 8.5, Ar-H), 7.16 (2H, d, *J* = 8.5, Ar-H), 7.00 (1H, s, Ar-H), 5.99–5.84 (1H, m, CH of allyl), 5.20 [1H, dd, *J* = 1.0 + 10.4, N-CH_2_ (H_a_)], 4.91 [1H, dd, *J* = 0.9 + 17.2, N-CH_2_ (H_b_)], 4.74 (2H, d, *J* = 4.9, =CH_2_), 4.55 (2H, s, CH_2_), 4.07 (2H, s, CH_2_), 2.83 (1H, hept, CH of isopropyl), 2.35 (6H, s, 2CH_3_), 1.17 (6H, d, *J* = 6.9, (CH_3_)_2_ of isopropyl).

#### 4.5.52 2-[5-(4,6-Dimethyl-pyrimidin-2-ylsulfanylmethyl)-4-(3-methoxy-propyl)-4*H*-[1,2,4]triazol-3-ylsulfanyl]-*N*-(4-isopropyl-phenyl)-acetamide (**47**)

Procedure as for **1a** except using **19** (212 mg, 1 mmol) and the reaction was carried out for 42 h. The precipitate formed was collected, washed with H_2_O and lyophilized, followed by recrystallization from EtOH. Pure **47** was obtained as a white powder (382 mg, 76%). *m*/*z* (ES), found 501.7603 (C_24_H_33_N_6_O_2_S_2_ [M+H]^+^) requires 501.2028; *δ*_H_/ppm (400 MHz, *d*^6^-DMSO): 10.21 (1H, s, NH), 7.43 (2H, d, *J* = 8.6, Ar-H), 7.16 (2H, d, *J* = 8.5, Ar-H), 7.00 (1H, s, Ar-H), 4.58 (2H, s, CH_2_), 4.10 (2H, s, CH_2_), 4.10 (2H, t, *J* = 7.2, *CH*_2_OCH_3_, this peak is partly covered by CH_2_ peak at 4.10), 3.28 (2H, t, *J* = 5.8, N-CH_2_), 3.20 (3H, s, OCH_3_), 2.83 (1H, hept, CH of isopropyl), 2.36 (6H, s, 2CH_3_), 1.98–1.87 (2H, m, CH_2_), 1.17 [6H, d, *J* = 6.9, (CH_3_)_2_ of isopropyl].

#### 4.5.53 2-[5-(4,6-Dimethyl-pyrimidin-2-ylsulfanylmethyl)-4-(4-methoxy-phenyl)-4*H*-[1,2,4]triazol-3-ylsulfanyl]-*N*-(4-isopropyl-phenyl)-acetamide (**48**)

A solution of **9** (360 mg, 1 mmol) in DMF (4 mL) and a solution of **19** (212 mg, 1 mmol) in DMF (2 mL) were combined in a dry Radleys reaction tube containing K_2_CO_3_ (166 mg, 1.2 mmol). The mixture was allowed to stir at 40 °C overnight in a Radleys parallel synthesizer. The mixture was poured on crushed ice (50 mL) and allowed to stand for few hours. The precipitate formed was collected by filtration, washed with H_2_O and lyophilized. The dried precipitate was washed with hot EtOH–MeOH (1:1) to afford **48** as a white powder (466 mg, 87%). *m*/*z* (ES), found 535.1592 (C_27_H_31_N_6_O_2_S_2_ [M+H]^+^) requires 535.1872; *δ*_H_/ppm (400 MHz, *d*^6^-DMSO): 10.22 (1H, s, NH), 7.45 (2H, d, *J* = 8.6, Ar-H), 7.35 (2H, d, *J* = 8.8, Ar-H), 7.17 (2H, d, *J* = 8.5, Ar-H), 6.98 (2H, d, *J* = 8.9, Ar-H), 6.91 (1H, s, Ar-H), 4.46 (2H, s, CH_2_), 4.09 (2H, s, CH_2_), 3.77 (3H, s, OCH_3_), 2.83 (1H, hept, CH of isopropyl), 2.26 (6H, s, 2CH_3_), 1.17 [6H, d, *J* = 6.9, (CH_3_)_2_ of isopropyl].

#### 4.5.54 2-[4-(4-Chloro-phenyl)-5-(4,6-dimethyl-pyrimidin-2-ylsulfanylmethyl)-4*H*-[1,2,4]triazol-3-ylsulfanyl]-*N*-(4-isopropyl-phenyl)-acetamide (**49**)

Procedure as for **1a** except using **19** (212 mg, 1 mmol). The precipitate formed was collected, washed with H_2_O, then lyophilized. The dried precipitate was washed with hot EtOH to afford **49** as a white powder (330 mg, 62%). *m*/*z* (ES), found 539.1047 (C_26_H_28_ClN_6_OS_2_ [M+H]^+^) requires 539.1376; *δ*_H_/ppm (400 MHz, *d*^6^-DMSO): 10.20 (1H, s, NH), 7.55–7.47 (4H, m, Ar-H), 7.43 (2H, d, *J* = 8.5, Ar-H), 7.17 (2H, d, *J* = 8.5, Ar-H), 6.92 (1H, s, Ar-H), 4.51 (2H, s, CH_2_), 4.08 (2H, s, CH_2_), 2.83 (1H, hept, CH of isopropyl), 2.27 (6H, s, 2CH_3_), 1.17 [6H, d, *J* = 6.9, (CH_3_)_2_ of isopropyl].

#### 4.5.55 2-[4-Allyl-5-(4,6-dimethyl-pyrimidin-2-ylsulfanylmethyl)-4*H*-[1,2,4]triazol-3-ylsulfanyl]-*N*-(4-trifluoromethyl-phenyl)-acetamide (**50**)

Procedure as for **1a** except using **20** (238 mg, 1 mmol). The brown precipitate formed was collected, washed with H_2_O and lyophilized, followed by recrystallization from EtOH. Pure **50** was obtained as a white powder (104 mg, 21%). *m*/*z* (ES), found 493.1109 (C_21_H_20_ F_3_N_6_OS_2_ [M−H]^−^) requires 493.1170; *δ*_H_/ppm (400 MHz, *d*^6^-DMSO): 10.64 (1H, s, NH), 7.74 (2H, d, *J* = 8.6, Ar-H), 7.70 (2H, d, *J* = 8.8, Ar-H), 6.99 (1H, s, Ar-H), 6.00–5.84 (1H, m, CH of allyl), 5.20 [1H, d, *J* = 10.4, N-CH_2_ (H_a_)], 4.92 (1H, d, *J* = 17.2, N-CH_2_ (H_b_)], 4.74 (2H, d, *J* = 4.9, =CH_2_), 4.55 (2H, s, CH_2_), 4.13 (2H, s, CH_2_), 2.35 (6H, s, 2CH_3_).

#### 4.5.56 2-[5-(4,6-Dimethyl-pyrimidin-2-ylsulfanylmethyl)-4-(3-methoxy-propyl)-4*H*-[1,2,4]triazol-3-ylsulfanyl]-*N*-(4-trifluoromethyl-phenyl)-acetamide (**51**)

Procedure as for **1a** except using **20** (238 mg, 1 mmol). The precipitate formed was collected, washed with H_2_O and lyophilisation, followed by recrystallization from EtOH. Pure **51** was obtained as a white powder (384 mg, 73%). *m*/*z* (ES), found 525.1343 (C_22_H_24_F_3_N_6_O_2_S_2_ [M−H]^−^) requires 525.1433; *δ*_H_/ppm (400 MHz, *d*^6^-DMSO): 10.65 (1H, s, NH), 7.74 (2H, d, *J* = 8.5, Ar-H) 7.67 (2H, d, *J* = 8.2, Ar-H), 7.00 (1H, s, Ar-H), 4.58 (2H, s, CH_2_), 4.16 (2H, s, CH_2_), 4.10 (2H, t, *J* = 6.7/7.6, *CH*_2_OCH_3_), 3.29 (2H, t, *J* = 5.8, N-CH_2_), 3.20 (3H, d, *J* = 2.2, O*CH*_3_), 2.35 (6H, s, 2CH_3_), 1.98–1.87 (2H, m, CH_2_).

#### 4.5.57 2-[5-(4,6-Dimethyl-pyrimidin-2-ylsulfanylmethyl)-4-(4-methoxy-phenyl)-4*H*-[1,2,4]triazol-3-ylsulfanyl]-*N*-(4-trifluoromethyl-phenyl)-acetamide (**52**)

Procedure as for **1a** except using **20** (238 mg, 1 mmol) and the reaction was carried out for 24 h. The precipitate formed was collected, washed with H_2_O, then lyophilized. The dried precipitate was washed with EtOH–MeOH (1:1) to afford **52** as a yellow powder (391 mg, 70%). *m*/*z* (ES), found 561.0706 (C_25_H_24_F_3_N_6_O_2_S_2_ [M+H]^+^) requires 561.1276; *δ*_H_/ppm (400 MHz, *d*^6^-DMSO): 10.67 (1H, s, NH), 7.76 (2H, d, *J* = 7.9, Ar-H), 7.68 (2H, d, *J* = 7.7, Ar-H), 7.36 (2H, d, *J* = 8.8, Ar-H), 6.99 (2H, d, *J* = 8.8, Ar-H), 6.91 (1H, s, Ar-H), 4.46 (2H, s, CH_2_), 4.15 (2H, s, CH_2_), 3.78 (3H, d, *J* = 1.6, OCH_3_), 2.27 (6H, s, 2CH_3_).

#### 4.5.58 2-[4-(4-Chloro-phenyl)-5-(4,6-dimethyl-pyrimidin-2-ylsulfanylmethyl)-4*H*-[1,2,4]triazol-3-ylsulfanyl]-*N*-(4-trifluoromethyl-phenyl)-acetamide (**53**)

Procedure as for **1a** except using **20** (238 mg, 1 mmol) and the reaction was carried out for 24 h. The precipitate formed was collected, washed with H_2_O and lyophilized. The dried precipitate was washed with EtOH–MeOH (1:1) to afford **53** as an off-white powder (460 mg, 81%). *m*/*z* (ES), found 565.0165 (C_24_H_20_ClF_3_N_6_OS_2_ [M+H]^+^) requires 565.0781; *δ*_H_/ppm (400 MHz, *d*^6^-DMSO): 10.65 (1H, s, NH), 7.75 (2H, d, *J* = 8.4, Ar-H), 7.68 (2H, d, *J* = 8.1, Ar-H), 7.56–7.45 (4H, m, Ar-H), 6.92 (1H, s, Ar-H), 4.51 (2H, s, CH_2_), 4.15 (2H, s, CH_2_), 2.26 (6H, s, 2CH_3_).

#### 4.5.59 *N*-(3,5-Bis-trifluoromethyl-phenyl)-2-[5-(4,6-dimethyl-pyrimidin-2-ylsulfanylmethyl)-4-(4-methoxy-phenyl)-4*H*-[1,2,4]triazol-3-ylsulfanyl]-acetamide (**54**)

Procedure as for **1a** except using **21** (306 mg, 1 mmol) and the reaction was carried out for 36 h. The precipitate formed was collected, washed with H_2_O and lyophilized. Without any further purification, **54** was obtained as a pale yellow powder (526 mg, 83%). *m*/*z* (ES), found 627.1069 (C_26_H_21_F_6_N_6_O_2_S_2_ [M−H]^−^) requires 627.1150; *δ*_H_/ppm (400 MHz, *d*^6^-DMSO): 10.97 (1H, s, NH), 8.21 (2H, s, Ar-H), 7.78 (1H, s, Ar-H), 7.36 (2H, d, *J* = 8.9, Ar-H), 6.99 (2H, d, *J* = 8.9, Ar-H), 6.90 (1H, s, Ar-H), 4.46 (2H, s, CH_2_), 4.14 (2H, s, CH_2_), 3.78 (3H, s, OCH_3_), 2.26 (6H, s, 2CH_3_).

#### 4.5.60 *N*-(4-Chloro-phenyl)-2-[5-(4,6-dimethyl-pyrimidin-2-ylsulfanylmethyl)-4-(4-methoxy-phenyl)-4*H*-[1,2,4]triazol-3-ylsulfanyl]-acetamide (**55**)

Procedure as for **1a** except using **23** (204 mg, 1 mmol) and the reaction was carried out for 42 h. The precipitate formed was collected, washed with H_2_O, and lyophilized. The dried precipitate was washed with EtOH–MeOH (1:1) to afford **55** as a white powder (462 mg, 88%). *m*/*z* (ES), found 527.0793 (C_24_H_24_ClN_6_O_2_S_2_ [M+H]^+^) requires 527.1012; *δ*_H_/ppm (400 MHz, *d*^6^-DMSO): 10.44 (1H, s, NH), 7.58 (2H, d, *J* = 8.9, Ar-H), 7.36 (2H, d, *J* = 8.9, Ar-H), 7.35 (2H, d, *J* = 8.9, Ar-H), 6.99 (2H, d, *J* = 8.9, Ar-H), 6.91 (1H, s, Ar-H), 4.46 (2H, s, CH_2_), 4.11 (2H, s, CH_2_), 3.78 (3H, s, OCH_3_), 2.26 (6H, s, 2CH_3_).

#### 4.5.61 2-[5-(4,6-Dimethyl-pyrimidin-2-ylsulfanylmethyl)-4-(4-methoxy-phenyl)-4*H*-[1,2,4]triazol-3-ylsulfanyl]-*N*-(4-methoxy-phenyl)-acetamide (**56**)

Procedure as for **1a** except using **22** (200 mg, 1 mmol) and the reaction was carried out for 42 h. The precipitate formed was collected, washed with H_2_O and lyophilized. The dried precipitate was washed with MeOH to afford **56** as a white powder (476 mg, 91%). *m*/*z* (ES), found 523.1320 (C_25_H_27_N_6_O_3_S_2_ [M+H]^+^) requires 523.1508; *δ*_H_/ppm (400 MHz, *d*^6^-DMSO): 10.16 (1H, s, NH), 7.45 (2H, d, *J* = 9.1, Ar-H), 7.35 (2H, d, *J* = 9.0, Ar-H), 6.99 (2H, d, *J* = 8.9, Ar-H), 6.91 (1H, s, Ar-H), 6.88 (2H, d, *J* = 9.1, Ar-H), 4.46 (2H, s, CH_2_), 4.08 (2H, s, CH_2_), 3.78 (3H, s, OCH_3_), 3.71 (3H, s, OCH_3_), 2.27 (6H, s, 2CH_3_).

#### 4.5.62 4-Furan-2-ylmethyl-5-methyl-4*H*-[1,2,4]triazole-3-thiol (**58**)

To a solution of **57** (4 g, 48.6 mmol) in EtOH (100 mL) was added furfuryl isothiocyanate (7.0 mL, 48.6 mmol) and the mixture was heated under reflux overnight. The reaction was cooled and concentrated under vacuum. The yellow precipitate obtained was washed with PE–EtOH (1:1). To the pale white precipitate thus obtained was added 2 M aq NaOH (30 mL) and the mixture was heated at 45 °C for 90 min before cooling. The reaction was neutralized with 10% aq HCl. The resulting white precipitate was washed with H_2_O and was lyophilized. Pure **58** was obtained as a white powder (3.7 g, 39%). *m*/*z* (ES), found 196.0995 (C_8_H_10_N_3_OS [M+H]^+^) requires 196.0466; *δ*_H_/ppm (400 MHz, *d*^6^-DMSO): 13.53 (1H, s, NH of thione tautomer), 7.62 (1H, t, *J* = 1.3/1.4, Ar-H), 6.43 (2H, d, *J* = 1.4, Ar-H), 5.22 (2H, s, CH_2_), 2.33 (3H, s, CH_3_).

#### 4.5.63 4-Allyl-5-methyl-4*H*-[1,2,4]triazole-3-thiol (**59**)

To a solution of **57** (4 g, 48.6 mmol) in EtOH (100 mL) was added allyl isothiocyanate (5 mL, 48.6 mmol) and the mixture was heated under reflux overnight. The reaction was cooled and concentrated under vacuum. The yellow precipitate obtained was washed with Et_2_O. To the white precipitate thus obtained was added 2 M aq NaOH (40 mL) and the mixture was heated at 45 °C for 60 min before cooling. The reaction was neutralized with 10% aq HCl. No precipitate was observed and the solution was allowed to stand overnight. The white crystalline solid thus formed was collected by filtration, washed with H_2_O and lyophilised. Pure **59** was obtained as a white crystalline solid (1.42 g, 19%). *m*/*z* (ES), found 156.1384 (C_6_H_10_N_3_S [M+H]^+^) requires 156.0517; *δ*_H_/ppm (400 MHz, *d*^6^-DMSO): 13.48 (1H, s, NH of thione tautomer), 5.94–5.79 (1H, m, CH), 5.19 [1H, dd, *J* = 1.3 + 10.4, N-CH_2_ (H_a_)], 5.00 [1H, dd, *J* = 1.3 + 17.2, N-CH_2_ (H_b_)], 4.60 (2H, d, *J* = 5.2, =CH_2_), 2.26 (3H, s, CH_3_).

#### 4.5.64 4-(3-Methoxy-propyl)-5-methyl-4*H*-[1,2,4]triazole-3-thiol (**60**)

To a solution of **57** (4 g, 48.6 mmol) in EtOH (100 mL) was added 3-methoxypropyl isothiocyanate (6.2 mL, 48.6 mmol) and the mixture was heated under reflux overnight. The reaction was cooled and concentrated under vacuum. A colourless oil was obtained, to which 1 M aq NaOH (20 mL) was added. The mixture was heated at 45 °C for 60 min, before cooling. The reaction was neutralized with 10% aq HCl. No precipitate was observed. The mixture was extracted with EtOAc, dried over NaSO_4_ and concentrated under vacuum. Pure **60** was obtained as a white powder (4.36 g, 48%). *m*/*z* (ES), found 188.0792 (C_7_H_14_N_3_OS [M+H]^+^) requires 188.0779; *δ*_H_/ppm (400 MHz, *d*^6^-DMSO): 13.40 (1H, s, NH of thione tautomer), 3.94 (2H, t, *J* = 7.3, *CH*_2_-OCH_3_), 3.33 (2H, t, *J* = 6.0, N-CH_2_), 3.23 (3H, s, OCH_3_), 2.30 (3H, s, CH_3_), 1.94–1.85 (2H, m, CH_2_).

#### 4.5.65 *N*-(3,4-Dimethyl-phenyl)-2-(4-furan-2-ylmethyl-5-methyl-4*H*-[1,2,4]triazol-3-ylsulfanyl)-acetamide (**62**)

A solution of **58** (195 mg, 1 mmol) in DMF (3 mL) and a solution of **13** (198 mg, 1 mmol) in DMF (2 mL) were combined in a dry Radleys reaction tube containing K_2_CO_3_ (166 mg, 1.2 mmol). The reaction mixture was allowed to stir at 45 °C overnight in a Radleys parallel synthesizer. The reaction mixture was poured on crushed ice (50 mL) and allowed to stand for few hours. The precipitate formed was collected by filtration, washed with H_2_O and lyophilized, followed by recrystallization from EtOH. Pure **62** was obtained as a white powder (208 mg, 58%). *m*/*z* (ES), found 356.9220 (C_18_H_21_N_4_O_2_S [M+H]^+^) requires 357.1307; *δ*_H_/ppm (400 MHz, *d*^6^-DMSO): 10.12 (1H, s, NH), 7.63 (1H, dd, *J* = 0.8 + 1.8, Ar-H), 7.32 (1H, d, *J* = 1.7, Ar-H), 7.26 (1H, dd, *J* = 2.0 + 8.1, Ar-H), 7.05 (1H, d, *J* = 8.2, Ar-H), 6.49 (1H, dd, *J* = 0.6 + 3.2, Ar-H), 6.44 (1H, dd, *J* = 1.8 + 3.2, Ar-H), 5.21 (2H, s, CH_2_), 4.04 (2H, s, CH_2_), 2.40 (3H, s, CH_3_), 2.18 (3H, s, CH_3_), 2.16 (3H, s, CH_3_).

#### 4.5.66 *N*-(3,4-Dimethyl-phenyl)-3-(4-furan-2-ylmethyl-5-methyl-4*H*-[1,2,4]triazol-3-ylsulfanyl)-propionamide (**63**)

Procedure as for **1a** except using **26** (423 mg, 2 mmol). The precipitate formed was collected, washed with H_2_O and lyophilized, followed by recrystallization from EtOH. Pure **63** was obtained as a white powder (89 mg, 12%). *m*/*z* (ES), found 369.1425 (C_19_H_21_N_4_O_2_S [M−H]^−^) requires 369.1463; *δ*_H_/ppm (400 MHz, *d*^6^-DMSO): 9.85 (1H, s, NH), 7.62 (1H, dd, *J* = 0.8 + 1.7, Ar-H), 7.34 (1H, d, *J* = 1.6, Ar-H), 7.27 (1H, dd, *J* = 2.0 + 8.1, Ar-H), 7.03 (1H, d, *J* = 8.2, Ar-H), 6.46–6.42 (2H, m, Ar-H), 5.26 (2H, s, CH_2_), 4.32 (2H, t, *J* = 7.3/7.4, CH_2_), 2.80 (2H, t, *J* = 7.3/7.4, CH_2_), 2.36 (3H, s, CH_3_), 2.17 (3H, s, CH_3_), 2.15 (3H, s, CH_3_).

#### 4.5.67 2-(4-Furan-2-ylmethyl-5-methyl-4*H*-[1,2,4]triazol-3-ylsulfanyl)-*N*-(4-isopropyl-phenyl)-acetamide (**64**)

Procedure as for **1a** except using **19** (212 mg, 1 mmol). The precipitate formed was collected, washed with H_2_O and lyophilized, followed by recrystallization from EtOH. Pure **64** was obtained as a white powder (181 mg, 49%). *m*/*z* (ES), found 370.9268 (C_19_H_23_N_4_O_2_S [M+H]^+^) requires 371.1463; *δ*_H_/ppm (400 MHz, *d*^6^-DMSO): 10.21 (1H, s, NH), 7.63 (1H, dd, *J* = 0.7 + 1.8, Ar-H), 7.45 (2H, d, *J* = 8.5, Ar-H), 7.17 (2H, d, *J* = 8.5, Ar-H), 6.48 (1H, d, *J* = 3.2, Ar-H), 6.44 (1H, dd, *J* = 1.8 + 3.2, Ar-H), 5.21 (2H, s, CH_2_), 4.05 (2H, s, CH_2_), 2.83 (1H, hept, CH of isopropyl), 2.40 (3H, s, CH_3_), 1.17 [6H, d, *J* = 6.9, (CH_3_)_2_].

#### 4.5.68 2-(4-Allyl-5-methyl-4*H*-[1,2,4]triazol-3-ylsulfanyl)-*N*-(4-isopropyl-phenyl)-acetamide (**65**)

Procedure as for **1a** except using **59** (155 mg, 1 mmol) and **19** (212 mg, 1 mmol). The precipitate formed was collected, washed with H_2_O and lyophilized, followed by recrystallization from EtOH. Product **65** was obtained as a white powder (143 mg, 43%). *m*/*z* (ES), found 330.9950 (C_17_H_23_N_4_OS [M+H]^+^) requires 331.1514; *δ*_H_/ppm (400 MHz, *d*^6^-DMSO): 10.19 (1H, s, NH), 7.44 (2H, d, *J* = 8.5, Ar-H), 7.17 (2H, d, *J* = 8.5, Ar-H), 6.00–5.85 (1H, m, CH of allyl), 5.19 [1H, dd, *J* = 1.1 + 10.4, N-CH_2_ (H_a_)], 4.88 [1H, dd, *J* = 1.1 + 17.2, N-CH_2_ (H_b_)], 4.59 (2H, d, *J* = 5.0, =CH_2_), 4.02 (2H, s, CH_2_), 2.83 (1H, hept, CH of isopropyl), 2.31 (3H, s, CH_3_), 1.17 [6H, d, *J* = 6.9, (CH_3_)_2_].

#### 4.5.69 *N*-(4-Isopropyl-phenyl)-2-[4-(3-methoxy-propyl)-5-methyl-4*H*-[1,2,4]triazol-3-ylsulfanyl]-acetamide (**66**)

Similar to **62** except using **60** (187 mg, 1 mmol) and **19** (212 mg, 1 mmol). The precipitate formed was collected, washed with H_2_O and lyophilized. Purification of the dried product by FCC [(EtOAc–MeOH (98:2)], gave product **66** as a white powder (120 mg, 33%). *m*/*z* (ES), found 362.9810 (C_18_H_27_N_4_O_2_S [M+H]^+^) requires 363.1776; *δ*_H_/ppm (400 MHz, *d*^6^-DMSO): 10.21 (1H, s, NH), 7.45 (2H, d, *J* = 8.5, Ar-H), 7.17 (2H, d, *J* = 8.5, Ar-H), 4.05 (2H, s, CH_2_), 3.95 (2H, t, *J* = 7.2/7.3, *CH*_2_-OCH_3_), 3.28 (2H, t, *J* = 5.8, N-CH_2_), 3.22 (3H, s, OCH_3_), 2.83 (1H, hept, CH of isopropyl), 2.34 (3H, s, CH_3_), 1.91–1.82 (2H, m, CH_2_), 1.17 [6H, d, *J* = 6.9, (CH_3_)_2_].
